# A RhoGEF activates RacM to selectively promote motility without affecting macropinocytosis in *Entamoeba histolytica*

**DOI:** 10.1242/jcs.264490

**Published:** 2026-06-16

**Authors:** Misato Shimoyama, Kumiko Nakada-Tsukui, Tomoyoshi Nozaki

**Affiliations:** ^1^ Department of Biomedical Chemistry, Graduate School of Medicine, The University of Tokyo, Tokyo, 113-0033, Japan; ^2^ Department of Parasitology, Japan Institute for Health Security, National Institute of Infectious Diseases, Tokyo 162-8640, Japan

**Keywords:** Rho small GTPase, RhoGEF, *Entamoeba histolytica*, Macropinocytosis, Migration

## Abstract

Rho guanine nucleotide-exchange factors (RhoGEFs) activate Rho and Rac small GTPases, orchestrating cellular processes, such as actin remodeling, endocytosis and migration. In the protozoan parasite *Entamoeba histolytica*, the causative agent of amebiasis, 22 Rho GTPases and ∼62 Dbl-homology (DH) domain-containing RhoGEFs have been identified, yet most remain uncharacterized. Our previous work revealed that *E. histolytica* (Eh)RacM (also known as EhRho13) negatively regulates macropinocytosis while promoting directional migration. Here, we characterize a previously uncharacterized RhoGEF, EHI_158230 (designated EhGEFM), identified as a potential EhRacM interactor through interactome analysis. Co-immunoprecipitation and *in vitro* GEF assays confirmed that EhGEFM directly binds and specifically activates EhRacM. Although *EhgefM* silencing did not affect macropinocytosis, it impaired directional migration, partially phenocopying *EhracM* silencing. Fixed and live-cell imaging revealed colocalization of EhGEFM and EhRacM at the cell periphery; notably, only EhRacM was recruited to maturing macropinosomes following actin coat disassembly. These findings indicate that although EhRacM is involved in both macropinocytosis and directional migration, its activator EhGEFM is required only for directional migration. This study provides the first direct evidence of distinct regulatory pathways governing Rho small GTPase function in *E. histolytica*.

## Teaser

**Summary:** *Entamoeba histolytica* (Eh)GEFM is identified as a RhoGEF of EhRacM, regulating migration but not macropinocytosis. This reveals the functional specificity of Rac signaling in this parasitic amoeba.

## INTRODUCTION

Rho guanine nucleotide-exchange factors (RhoGEFs) play a crucial role in the regulation of Rho and Rac (hereafter referred to as Rho) small GTPases, which are members of the Ras superfamily and serve as key conserved regulators of the actin cytoskeleton. RhoGEFs catalyze the exchange of GDP for GTP, thereby converting Rho GTPases from their inactive to active forms. This activation is essential for the signal transduction pathways governing diverse cellular processes, including actin cytoskeleton remodeling, cell-cycle progression, transcriptional regulation, endocytosis and cell migration (Rossman et al., 2005). The Dbl-homology (DH) domain-containing proteins represent the largest class of RhoGEFs (Rossman et al., 2005; Schmidt and Hall, 2002). The DH domain facilitates GDP release from Rho GTPases through a ‘push-and-pull’ mechanism: in the ‘push’ phase, the DH domain engages with the switch I region (G2 box), disrupting Mg^2+^ coordination; in the subsequent ‘pull’ phase, conformational rearrangements in the switch II region (G3 box) further facilitate GDP release (Rossman et al., 2005). Importantly, the DH domain-Rho GTPase interaction exhibits substrate specificity, allowing certain Dbl proteins to selectively activate distinct Rho GTPases (Rossman et al., 2005).

*Entamoeba histolytica* is an enteric protozoan parasite and the etiological agent of amebiasis, a major infectious disease responsible for ∼55,000 deaths annually worldwide, especially in low- and middle-income countries (Shirley et al., 2018). The genome of *E. histolytica* encodes 22 Rho GTPases [denoted *E. histolytica* (Eh)Rho and EhRac proteins] (Chung et al., 2010), several of which have been functionally characterized in the context of its pathogenesis. For example, EhRho1B (EHI_029020; referred to as EhRho1 in Bharadwaj et al., 2018; Chung et al., 2010) recruits the actin-binding proteins EhFormin1 and EhProfilin1 during phagocytosis (Bharadwaj et al., 2018). Constitutively active EhRacA2 (EHI_197840; refereed to as EhRacA in Ghosh and Samuelson, 1997, and also known as EhRho6; Chung et al., 2010; Narooka et al., 2022) disrupts erythrophagocytosis and impairs surface receptor capping, an immune evasion mechanism (Ghosh and Samuelson, 1997). Similarly, constitutively active EhRacG (EHI_129750; also known as EhRho2; Chung et al., 2010; Narooka et al., 2022) impairs cytokinesis, induces multinucleation and alters the cell polarity of trophozoites by promoting F-actin condensation at one end of the cell (Guillén et al., 1998).

The *E. histolytic*a genome encodes ∼62 predicted DH-domain-containing RhoGEFs (EhGEFs), a number comparable to the 69 found in the human genome (Vargas and González-de la Rosa, 2007). However, functional characterization of these proteins remains limited. EhGEF1 (EHI_049610) has been proposed to regulate EhRho1B (Franco-Barraza et al., 2006) and to participate in actin dynamics, cytokinesis, capping and uroid formation (Aguilar-Rojas et al., 2005). EhGEF2 (EHI_182740) has been shown to preferentially activate EhRacG and implicated in erythrophagocytosis, cell proliferation, and chemotaxis (González De la Rosa et al., 2007). The EhGEF3 (EHI_098820)–EhRacA2 axis might regulate uroid retraction and chemotactic responses to fibronectin (Arias-Romero et al., 2007). Another RhoGEF, EhFP4 (EHI_153510), which contains a Fab-1, YGL023, Vps27 and EEA1 (FYVE) domain and binds to phosphatidylinositol 4-phosphate [PtdIns(4)P], has been suggested to play a role in trogocytosis (Nakada-Tsukui et al., 2009).

In our previous study, we identified a Rho small GTPase, EhRacM (EHI_135450; also known as EhRho13; Chung et al., 2010; Narooka et al., 2022), as a negative regulator of macropinocytosis and a positive regulator of directional migration (Shimoyama et al., 2024). From an interactome analysis, we identified a DH domain-containing protein, EHI_158230, as a candidate interacting partner of EhRacM. This protein, which we designate here as EhGEFM, has not yet been functionally characterized. In this study, we performed a functional analysis of EhGEFM, confirming its physical interaction with EhRacM by co-immunoprecipitation and demonstrating its substrate specificity via an *in vitro* RhoGEF assay. Although *EhgefM* silencing did not alter macropinocytosis, it impaired directional migration, partly mimicking the effects observed in *EhracM* gene silencing. Live-cell and fixed-cell imaging showed colocalization of EhGEFM and EhRacM at the cell periphery, whereas only EhRacM was recruited to maturing macropinosomes following actin coat disassembly. The recruitment of EhRacM to macropinosomes might involve a negative signal for macropinocytosis that is independent of EhGEFM. These findings suggest that EhRacM mediates distinct signaling pathways and that EhGEFM functions specifically in directional migration.

## RESULTS

### Interactome analysis of EhGEFM confirmed its interaction with EhRacM

To explore how Rho GTPase signaling is coordinated in *E. histolytica*, we focused on EhGEFM, which was initially identified in the interactome of EhRacM (Shimoyama et al., 2024). We began by performing a reciprocal interactome analysis to validate this interaction. To identify the binding partners of EhGEFM, HA-tagged EhGEFM (HA–EhGEFM) was overexpressed in *E. histolytica* trophozoites (see Fig. 4A), followed by co-immunoprecipitation (co-IP) using anti-HA antibody-conjugated agarose beads. The co-precipitated proteins were subsequently subjected to mass spectrometry-based proteomic analysis. Mass spectrometry confirmed peptides spanning most of the protein sequence, excluding only the extreme N- and C-terminal regions, without evidence of truncation (Fig. S1A,B).

In this analysis, we selected candidate proteins (hits) with QV values exceeding one-hundredth of the bait protein (EhGEFM) and consistently enriched (>1-fold) compared to the mock control in two independent experiments. Using these criteria, we identified 168 candidates (Table S3). Among them, EhRacM (EHI_135450) was detected exclusively in lysates from HA–EhGEFM-overexpressing cells, but not in the mock control, supporting the proposed interaction. Unlike EhRacM, other Rhos, such as EhRacG (EHI_129750; Chung et al., 2010) and EhRacQ (EHI_146180; Chung et al., 2010), were also detected in both samples with only modest (<2-fold) enrichment (Table S3).

To further characterize these hits, we performed Gene Ontology (GO) enrichment analysis. This analysis revealed a strong overrepresentation of biological processes related to ribosome assembly, ribonucleoprotein complex biogenesis, translation and cellular component organization in HA–EhGEFM-overexpressing cells (Fig. S1C). Molecular function enrichment highlighted ribosomal subunit binding, RNA binding and structural constituents of the ribosome, alongside broader categories such as protein binding and actin binding (Fig. S1D). According to the PANTHER protein class, 30 hits were ribosomal proteins. The actin-binding proteins contained C-terminal filamentous actin (F-actin)-bundling proteins (EHI_010570, EHI_004550 and EHI_086690), SH3-domain-containing proteins (EHI_054800 and EHI_180030), WH2-domain-containing proteins (EHI_007000 and EHI_016130), a calponin homology domain protein (EHI_199000), coronin (EHI_083590), filopodin (EHI_167130), the Dot/Icm system substrate protein LidA (EHI_083620) and RasGAP (EHI_035800). Notably, 123 out of the 168 hits had also been previously identified in the EhRacM interactome in at least one of three independent experiments (Shimoyama et al., 2024) (Table 1; Table S3). Table 1 highlights the hits that were also detected across all three HA–EhRacM interactome analyses. These include adaptor protein (AP)-related proteins (EHI_083430, EHI_013040 and EHI_164810), proteasome-related proteins (EHI_078710, EHI_194570, EHI_179970, EHI_164750 and protease regulatory subunit, putative), and G-protein signaling/small GTPase-related proteins (EHI_135450, EHI_140350, EHI_147570 and EHI_146180). Of note, cortexillin, which is a coiled-coil actin-bundling protein originally identified in *Dictyostelium discoideum* (Faix et al., 1996), was exclusively detected. It organizes actin filaments into antiparallel bundles, contributing to cortical actin organization and cytokinesis (Faix et al., 1996).

**Table 1.** Hits commonly detected in the HA–EhGEFM and HA–EhRacM interactomes

Accession Number	Description	Mean QV of mock	Mean QV of HA–EhGEFM	QV ratio (HA–EhGEFM/mock)
**EHI_158230**	**DH domain-containing protein (EhGEFM)**	**3.80**	**328.12**	**86.35**
EHI_135450	Rho family GTPase (EhRacM)	0	19.20	-
EHI_191900	Cortexillin, putative	0	8.43	-
EHI_083430	Adaptor-related protein complex 3 (AP-3) subunit, putative	1.20	5.93	4.96
EHI_078710	Proteasome subunit beta	1.20	5.22	4.37
EHI_148170	Serine/threonine-protein phosphatase 2A 55 kDa regulatory subunit B	0.96	4.18	4.36
EHI_004640	Vesicle-fusing ATPase	2.15	8.11	3.76
EHI_124870	HEAT repeat domain containing protein	2.09	6.93	3.31
EHI_013040	Adaptor protein (AP) family protein	2.81	6.72	2.39
EHI_194570	26S protease regulatory subunit, putative	3.95	9.40	2.38
EHI_164810	AP-3 complex subunit delta	4.27	9.29	2.17
EHI_179970	Proteasome regulatory subunit, putative	4.66	8.95	1.92
EHI_110690	Variant sh3 domain containing protein	2.99	5.59	1.87
EHI_051710	60S ribosomal protein L23, putative	5.89	9.24	1.57
EHI_140350	G protein alpha subunit, putative	3.97	5.33	1.34
EHI_164750	26S proteasome non-ATPase regulatory subunit 14, putative	3.71	4.96	1.34
EHI_185410	26S protease regulatory subunit, putative	10.40	13.76	1.32
EHI_198740	40S ribosomal protein S19, putative	11.42	14.87	1.30
EHI_147570	Rho GDP exchange inhibitor, putative	4.60	5.15	1.12
EHI_146180	Rho family GTPase (EhRacQ)	5.08	5.67	1.12
EHI_047800	Uncharacterized protein	8.16	8.61	1.06

Hits shown were detected in all co-IP experiments (in two HA–EhGEFM and three HA–EhRacM interactome analyses).

### Structural features of EhGEFM

Next, to gain insights into the structural features of EhGEFM, we analyzed its amino acid sequence using InterPro. This analysis revealed the presence of three conserved domains: an armadillo (Arm)-like helical [amino acids (aa) 58–384], DH domain (aa 415–596), and PH domain (aa 600–737) in EhGEFM (Fig. S2A–D). The PH domains are frequently found adjacent to DH domains in RhoGEFs and are proposed to contribute to their membrane localization and regulation of GEF activity through interactions with phosphoinositides (Rossman et al., 2005). Notably, the presence of Arm repeats is uncommon among EhGEFs; only four out of 62 predicted DH domain-containing EhGEFs are predicted to possess these repeats. Arm repeats are typically involved in mediating protein–protein interactions (Andrade et al., 2001). In EhGEF2 (EHI_182740), which also contains Arm repeats, these motifs have been implicated in both proper subcellular localization and catalytic activity (González De la Rosa et al., 2007).

We further confirmed the presence of conserved regions (CR1–CR3) within the DH domain of EhGEFM (Fig. S2E). It is known that the switch I region of Rho small GTPases interacts with CR1 and CR3, whereas the switch II region predominantly contacts CR3 and portions of the C-terminal helix contact (Rossman et al., 2005). Specifically, EhGEFM harbors threonine 426 (T426) and an asparagine–glutamate pair at positions 594–595 (N594–E595), which are key residues directly involved in the catalytic activity of DH RhoGEFs (Aghazadeh et al., 1998; Debreceni et al., 2004; Zhu et al., 2000).

In addition, AlphaFold2 multimer modelling of full-length EhGEFM in complex with EhRacM predicted a canonical DH–switch I/II interface, with EhGEFM CR1 and CR3 positioned to engage the Switch I (P42–D46) and switch II (G68–R74) regions of EhRacM (Fig. S7C,D). Notably, the model also suggested secondary contacts between the Arm domain of EhGEFM and residues S130–L135 of EhRacM (Fig. S7C,D), located within the Rho insert region (Shimoyama et al., 2024). Although these Arm–Rac interactions are unlikely to contribute directly to guanine nucleotide exchange, their spatial positioning could influence the relative orientation of the DH domain and thereby modulate catalytic efficiency.

### EhGEFM exhibited guanine nucleotide exchange activity toward EhRacM

After confirming the interaction between EhRacM and EhGEFM, we next investigated whether EhGEFM functions as an upstream RhoGEF for EhRacM by assessing its guanine nucleotide exchange activity *in vitro*. We produced N-terminal histidine (His)-tagged recombinant EhGEFM using the *E. coli* pCold I expression system: the full-length (FL) protein [His–EhGEFM (FL)] and a truncated variant lacking the Arm repeats (His–EhGEFMΔArm; aa 391–738). The truncated construct was designed to assess the functional contribution of the Arm repeats to RhoGEF activity. SDS-PAGE followed by Coomassie Brilliant Blue (CBB) staining confirmed that both recombinant proteins were highly pure and corresponded to their predicted molecular masses (88.4 kDa for His–EhGEFM (FL) and 44.9 kDa for His–EhGEFMΔArm) (Fig. S3A,B). In parallel, we produced and purified five recombinant EhRho or EhRac proteins in *E. coli* as N-terminal glutathione S-transferase (GST) fusion proteins using the pCold-GST system. These included EhRacM, EhRacA2 (Chung et al., 2010), EhRacD1 (EHI_012240; also known as EhRho5; Chung et al., 2010; Narooka et al., 2022), EhRacG (Chung et al., 2010) and EhRho1B (Chung et al., 2010) (Table S1). SDS-PAGE followed by CBB staining confirmed that the recombinant GST–EhRho or Rac proteins were highly pure and migrated at their expected molecular masses (51.1, 50.2, 51.0, 50.8 and 52.4 kDa, respectively) (Fig. S3C–G).

**Fig. 1.**
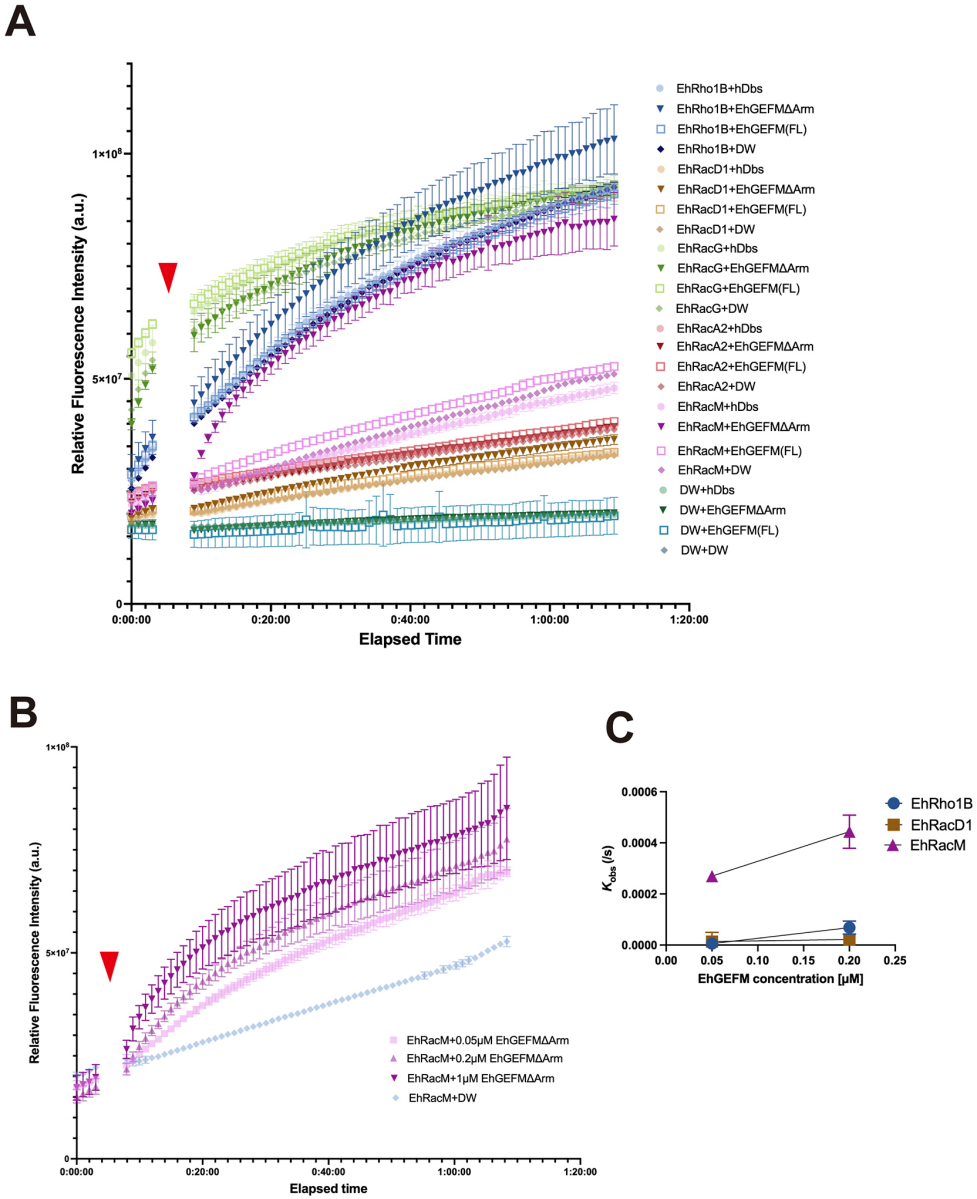
**Evaluation of RhoGEF activity of EhGEFM toward EhRacM.** Relative fluorescence intensity was measured using a plate reader. (A) After four initial readings, either hDbs–His, His–EhGEFM (FL), His–EhGEFMΔArm, or distilled water (DW) was added (indicated by the red triangle) to the respective Rho small GTPase (GST–EhRacM, GST–EhRacA2, GST–EhRacD1, GST–EhRacG or GST–EhRho1B), and readings were resumed. (B) After four initial readings, either the indicated concentration of His–EhGEFMΔArm or DW was added (indicated by the red triangle) to GST–EhRacM. (C) Catalytic efficiency (*k*_cat_*/K*_m_) of EhGEFM against EhRho1B, EhRacD1 and EhRacM was obtained from the slope of a linear least square fit of *K*_obs_ values against EhGEFM concentration (for EhRacM, see B). Data in A to C represent the mean±s.d. of three technical replicates. a.u., arbitrary units.

Subsequently, to assess the RhoGEF activity of EhGEFM toward these EhRho and EhRacs, we employed an N-MAR-GTP fluorophore-based RhoGEF exchange assay. The assay setup was validated by measuring the RhoGEF activities of His tag-conjugated human RhoGEF Dbs (hDbs) toward His tag-conjugated human Cdc42, Rac1 and RhoA (Fig. S3H). Although each EhRho or EhRac exhibited intrinsic GTPase activity in the absence of RhoGEF, the addition of His–EhGEFMΔArm significantly enhanced nucleotide exchange specifically for GST–EhRacM (Fig. 1A). A modest increase in nucleotide exchange was also observed for EhRho1B. However, these effects were not detected when full-length His–EhGEFM was used (Fig. 1A). To further assess the specificity and catalytic activity of EhGEFM, we determined the observed rate constants (*K*_obs_) of nucleotide exchange for EhRacM, EhRho1B and EhRacD1 at increasing concentrations of His–EhGEFMΔArm. Catalytic efficiency was calculated from the slope of the linear least squares fit of the *K*_obs_ values plotted against the EhGEFM concentration (Apte et al., 2022). This assay confirmed EhRacM as the preferred substrate, with a catalytic efficiency of 1160 M^−1^ s^−1^ (Fig. 1B,C). In contrast, EhRho1B and EhRacD1 exhibited lower catalytic efficiencies (418 M^−1^ s^−1^ and 54 M^−1^ s^−1^, respectively) (Fig. 1C).

### Gene silencing of *EhgefM* did not affect macropinocytosis

**Fig. 2.**
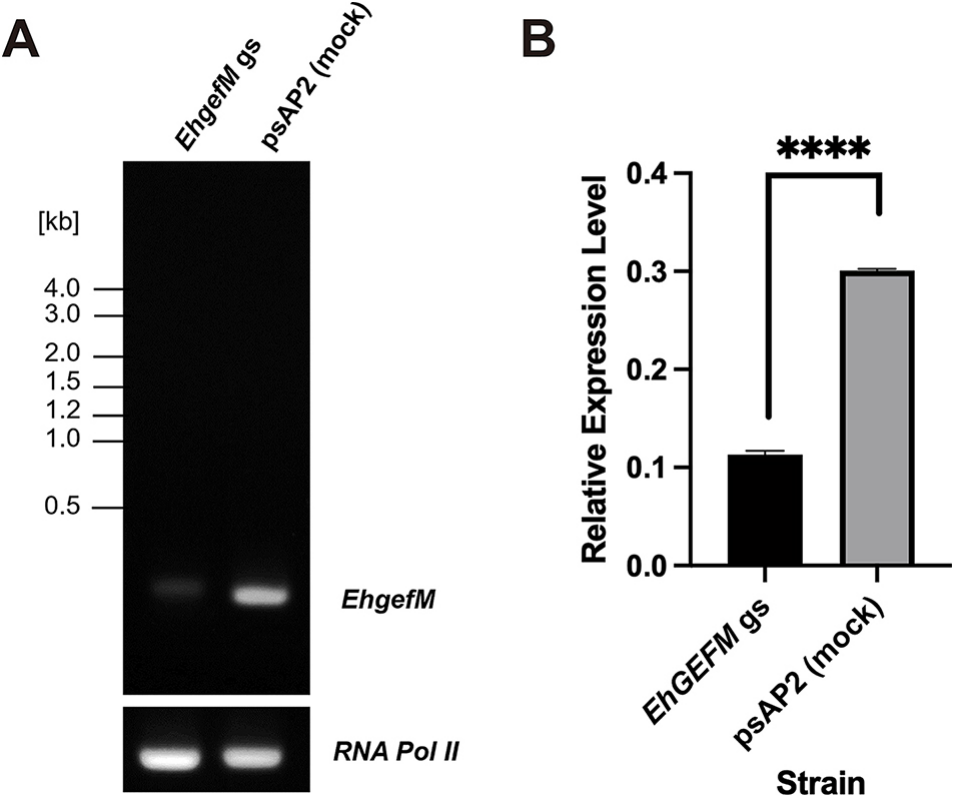
**Gene silencing of *EhgefM.*** Expression silencing was confirmed by RT-PCR (estimated size of *EhgefM*, 159 bp; RNA Pol II, 182 bp) (A) and qRT-PCR (B). (A) Representative image of agarose gel electrophoresis is shown. (B) Average relative expression level of *EhGEFM* normalized to RNA Pol II mRNA, as measured by qRT-PCR. Data are mean±s.d. from three technical replicates. ****P<0.0001 (unpaired two-tailed *t*-test). gs, gene silenced.

**Fig. 3.**
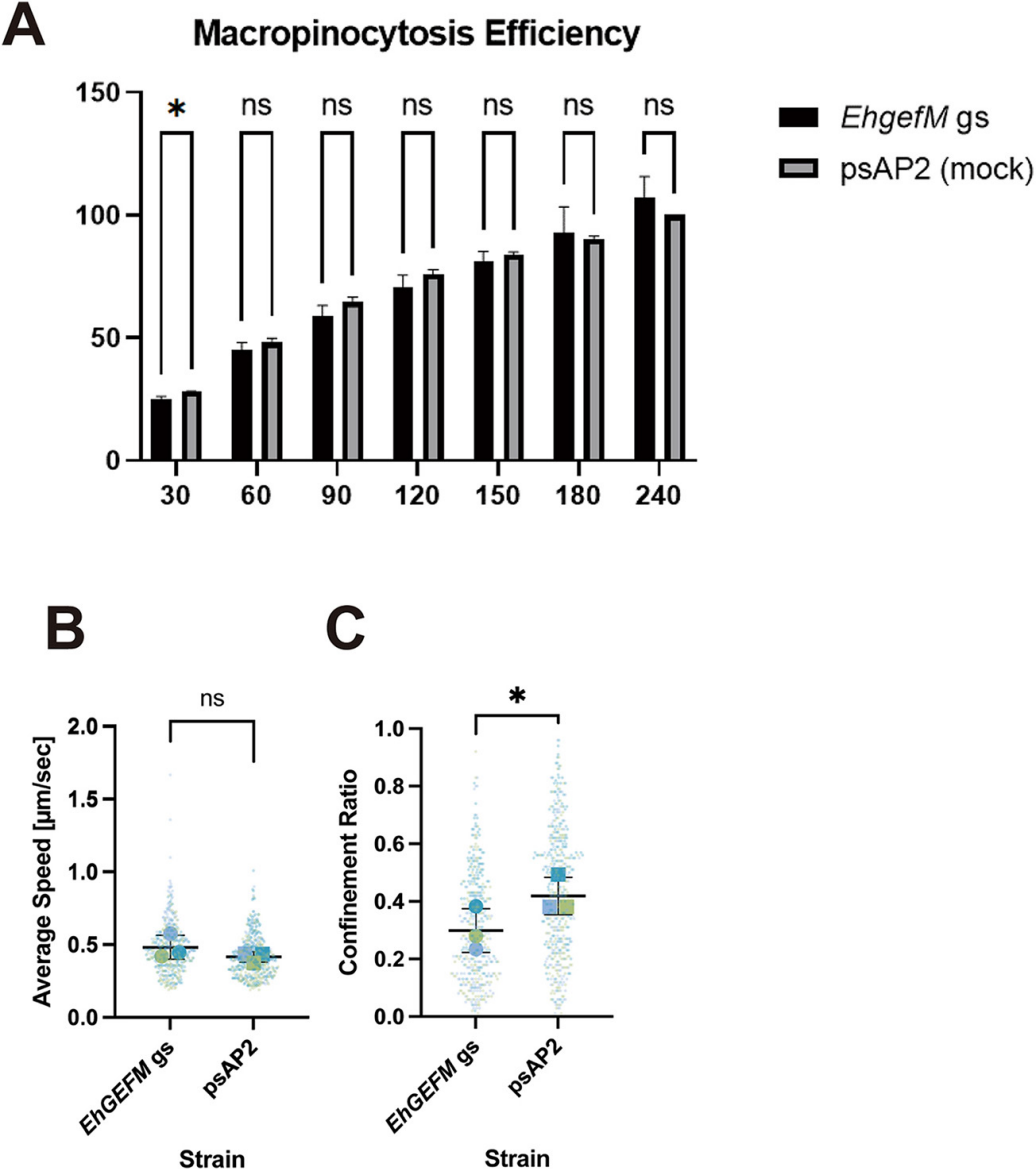
**Phenotypic assays of the *EhgefM* gene-silenced strain.** (A) Macropinocytosis of *EhgefM* gene-silenced (gs) and psAP2 mock control strains. Trophozoites were incubated in RITC–dextran-containing BIS medium, and macropinocytosis was quantified by FACS. The RITC–dextran incorporation of each strain was estimated by calculating the geometric mean of the fluorescence intensity after the background signal from unlabeled parasites was subtracted from the fluorescence intensity in the PE-A channel of each strain, and is shown relative to the value of the mock strain at 240 min. Data are mean±s.d. of three biological replicates. (B,C) Results of the motility assay analyzed by CQ1, representing average speed (B) and confinement ratio (C). Each small, semi-transparent dot corresponds to the motility value of a single trophozoite, color-coded by biological replicate. Larger dots indicate the mean value of each replicate, using the same color scheme. Approximately 200 trophozoites were analyzed per biological replicate. Bars represent the mean±s.d. of three biological replicates. *P<0.05; ns, not significant (unpaired two-tailed *t*-test).

Given that silencing of *EhracM* results in an upregulation of macropinocytosis (Shimoyama et al., 2024), we hypothesized that *EhgefM* silencing might produce a similar phenotype if EhGEFM functions as an upstream regulator of EhRacM. To test this, we established an *EhgefM* gene-silenced strain using antisense small RNA-mediated transcriptional gene silencing (Mi-ichi et al., 2011; Penuliar et al., 2012). We examined the repression of the *EhgefM* gene expression by reverse transcriptase (RT)-PCR (Fig. 2A) and quantitative real-time (qRT)-PCR (Fig. 2B), confirming a significant reduction in gene expression compared to that in the mock strain. Note that the basal expression level of *EhgefM* is relatively low compared to other EhGEFs that have been studied so far (Fig. S2F). To investigate the functional impact of *EhgefM* silencing on macropinocytosis, we quantified fluid-phase uptake using a flow cytometry-based assay. In this assay, amoebic trophozoites were incubated with Rhodamine B isothiocyanate (RITC)-conjugated dextran, and dextran uptake was quantified by measuring fluorescence intensity increase. In *E. histolytica*, fluid-phase uptake is generally considered to occur predominantly via actin-dependent macropinocytosis (Movie 1), which enables bulk fluid-phase uptake, although clear experimental criteria to distinguish it from other endocytic routes remain limited. Contrary to our expectations, *EhgefM* gene silencing did not produce a significant effect on macropinocytosis (Fig. 3A).

**Fig. 4.**
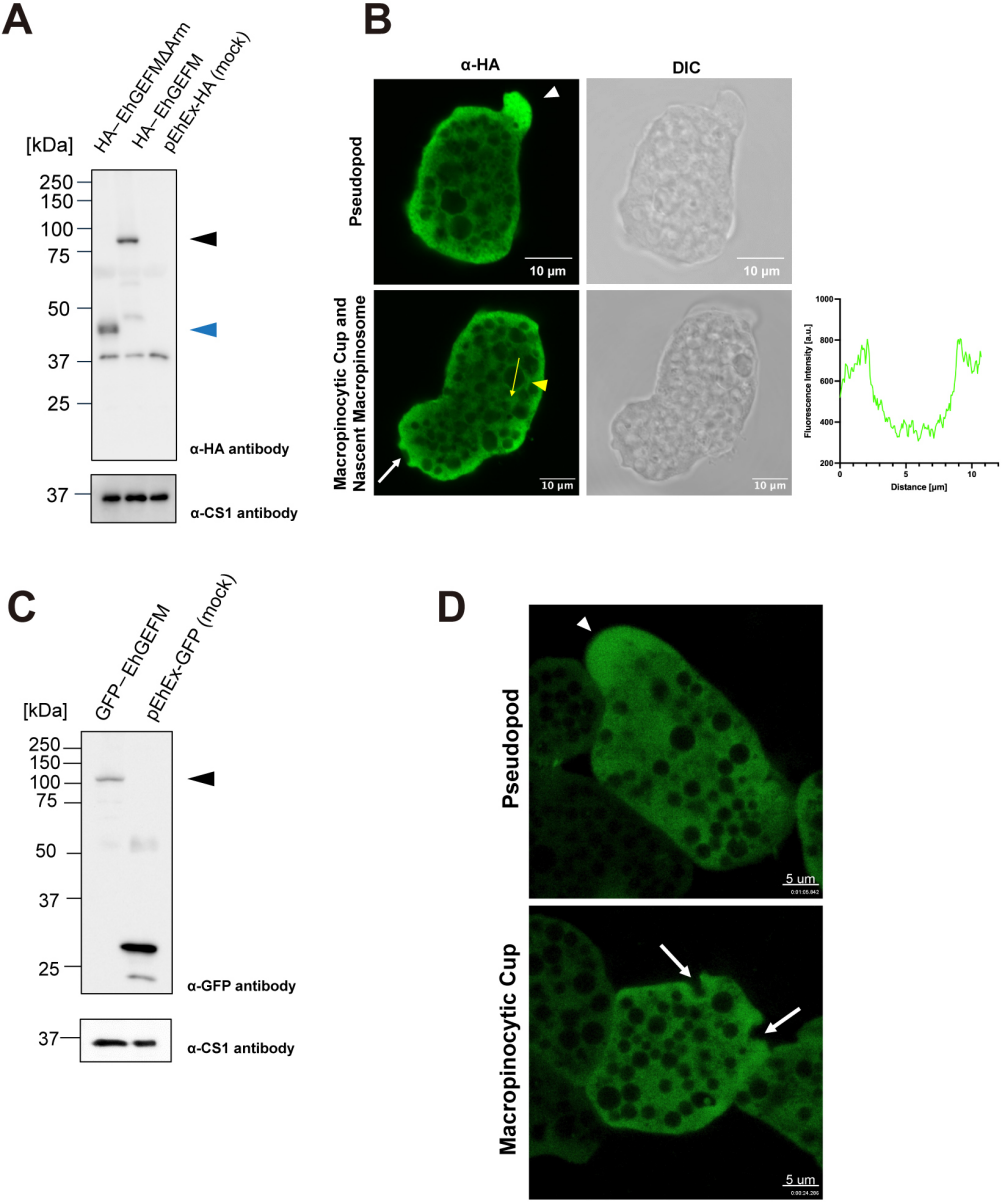
**Localization of HA– or GFP–EhGEFM.** (A) Immunoblot detection of HA–EhGEFM and HA–EhGEFMΔArm in *E. histolytica* transformants. Approximately 30 μg of total lysates from HA-fused EhGEFM or EhGEFMΔArm expressing transformants and mock-transfected control (pEhEx-HA) were subjected to SDS-PAGE followed by immunoblot analysis using anti-HA monoclonal antibody and anti-CS1 polyclonal antibody (loading control). The black arrowhead indicates the approximate size of HA–EhGEFM, whereas the blue arrowhead indicates the approximate size of HA–EhGEFMΔArm. Blot shown representative of two repeats. (B) Immunofluorescence images of *E. histolytica* trophozoites from the HA–EhGEFM-overexpressing strain. The left panels show HA–EhGEFM visualized using an anti-HA antibody, whereas the right panels display the corresponding DIC images. The upper panels depict a trophozoite with HA–EhGEFM enrichment at the pseudopod (white arrowhead), whereas the lower panels show a trophozoite with concentrated HA–EhGEFM at the macropinocytic cup (white arrow) as well as the nascent macropinosome surface (yellow arrowhead). The line plot indicates the fluorescence intensity along the yellow arrow. Images representative of multiple events observed in one experiment. Scale bars: 10 μm. (C) Immunoblot detection of GFP–EhGEFM in *E. histolytica* transformants. Approximately 30 μg of total lysates from GFP-fused EhGEFM expressing transformants and mock-transfected control (pEhEx-GFP) were subjected to SDS-PAGE followed by immunoblot analysis using anti-GFP monoclonal antibody and anti-CS1 polyclonal antibody (loading control). The black arrowhead indicates the approximate size of GFP–EhGEFM. Blot is from one experiment. (D) Representative *E. histolytica* trophozoites from the live imaging of the GFP–EhGEFM strain. The upper panels show a trophozoite with concentrated GFP–EhGEFM at a pseudopod (white arrowhead), whereas the lower panels depict a trophozoite with GFP–EhGEFM enrichment at the macropinocytic cups (white arrows). Images representative of multiple events observed in one experiment. Scale bars: 5 μm.

### *EhgefM* silencing impaired directional persistence during cell migration

As *EhracM* gene silencing has been shown to impair directional persistence during trophozoite migration (Shimoyama et al., 2024), potentially through disruption of cytoskeletal organization or signaling pathways, we investigated whether gene silencing of *EhgefM* also produces a similar phenotype. To this end, we analyzed the motility of the *EhgefM* gene-silenced trophozoites using time-lapse imaging (Movie 2), quantifying average speed and confinement ratio, as described previously (Shimoyama et al., 2024). Here, average speed reflects the overall activity or motility of cells, and the confinement ratio (calculated as net distance/total distance) represents directional persistence (Allier et al., 2014; Krause and Gautreau, 2014). We observed a significant reduction in confinement ratio in the *EhgefM* gene silenced strain (Fig. 3C), indicating impaired directional persistence (Maiuri et al., 2015). Interestingly, average speed remained unchanged compared to the mock control (Fig. 3B). Notably, this phenotype closely mirrors that observed in the *EhracM* gene-silenced strain.

### Expression of Arm repeat-deleted EhGEFM slightly affects cell motility and macropinocytosis

To further examine the role of EhGEFM in macropinocytosis and cell migration, we generated *E. histolytica* trophozoites expressing Arm repeat-deleted mutant of EhGEFM (HA–EhGEFMΔArm). Based on the *in vitro* GEF assay described above, deletion of the Arm repeats was expected to relieve the autoinhibitory effect and generate a constitutively active GEF variant.

Expression of HA–EhGEFMΔArm was confirmed by immunoblotting at the expected molecular mass of ∼45 kDa (41.5 kDa for EhGEFMΔArm plus three 1.1 kDa HA tags) (Fig. 4A). During cultivation, we noticed that the HA–EhGEFMΔArm-overexpressing strain grew more slowly than the mock control and the HA–EhGEFM-overexpressing strain. The doubling times of the mock control, HA–EhGEFM, and HA–EhGEFMΔArm strains were 12.8, 13.3 and 20.7 h, respectively, indicating that HA–EhGEFMΔArm cells proliferated ∼1.6-fold more slowly than the other strains. Immunofluorescence analysis showed that HA–EhGEFMΔArm localized diffusely throughout the cytoplasm with occasional enrichment at pseudopods (Fig. S4A).

We next examined whether Arm repeat deletion would affect macropinocytosis. Fluid-phase uptake assays showed a slight increase in dextran uptake in the HA–EhGEFMΔArm strain compared with that in the mock control or HA–EhGEFM-overexpressing strain; however, the difference was not statistically significant (Fig. S4B). Finally, we analyzed cell motility using time-lapse imaging. Although the average speed of HA–EhGEFMΔArm cells was comparable to that of the mock strain (Fig. S4C), confinement ratio tended (not statistically significant) to increase, compared to that of mock and HA–EhGEFM-overexpressing strains, suggesting more persistent directional movement (Fig. S4D).

Taken together, these results suggest that expression of Arm repeat-deleted EhGEFM slightly increases dextran macropinocytosis and directional cell migration. The tendency observed in the motility assay contrasts with the phenotype of the *EhgefM* gene-silenced strain, which showed reduced directional persistence.

### Subcellular localization and dynamics of EhGEFM in *E. histolytica* trophozoites

**Fig. 5.**
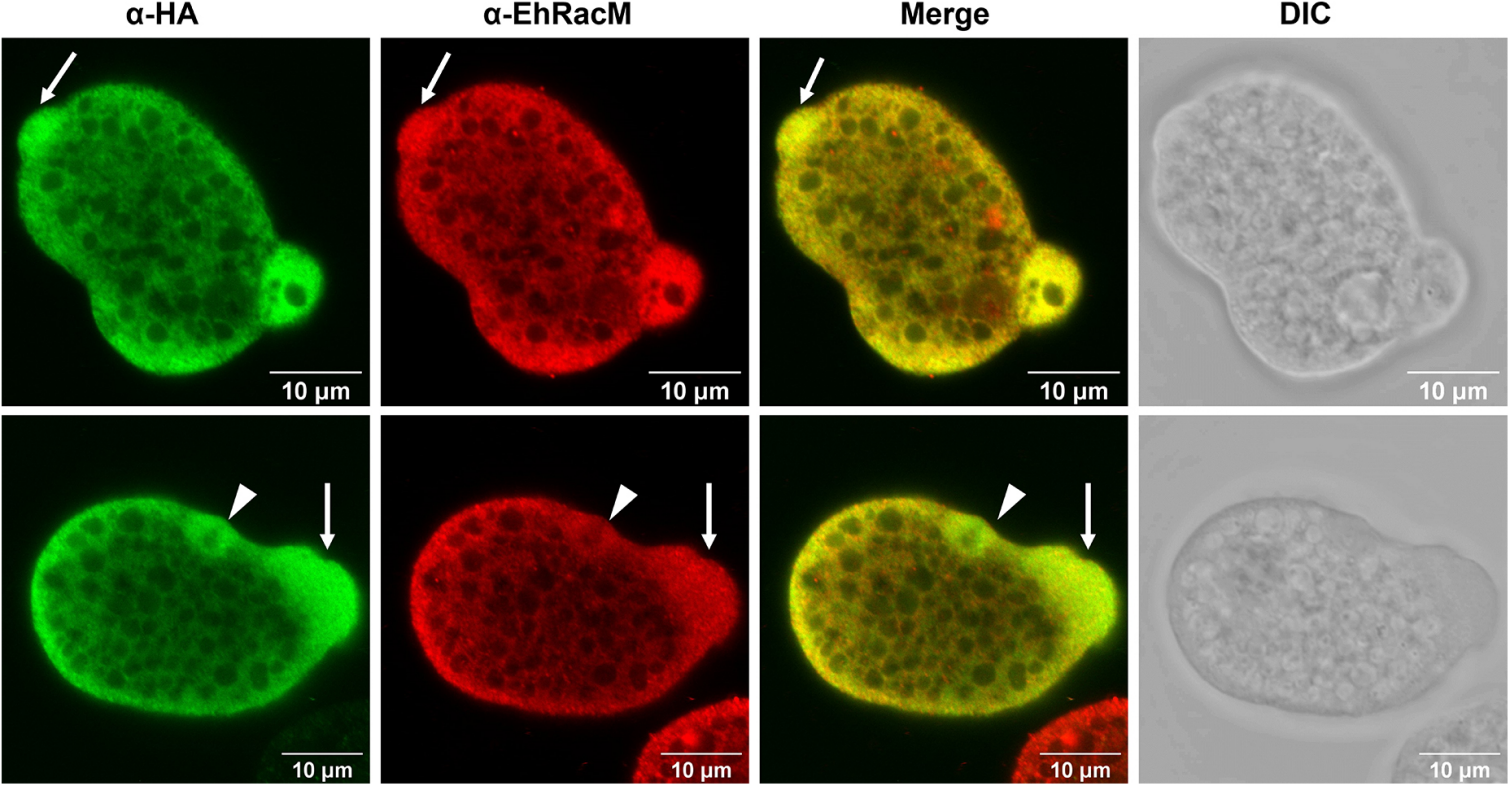
**Localization of HA–EhGEFM and EhRacM in HA–EhGEFM-overexpressing *E. histolytica* trophozoites.** The first column shows HA–EhGEFM visualized using an anti-HA antibody (green), and the second column shows EhRacM stained with an anti-EhRacM antibody (red). The third column shows merged images of the anti-HA and anti-EhRacM staining, and the fourth column displays the corresponding DIC images. Arrowheads point to a possible nascent macropinosome with HA–EhGEFM accumulation. Arrows indicate pseudopods. Images representative of two biologically independent repeats. Scale bars: 10 μm.

**Fig. 6.**
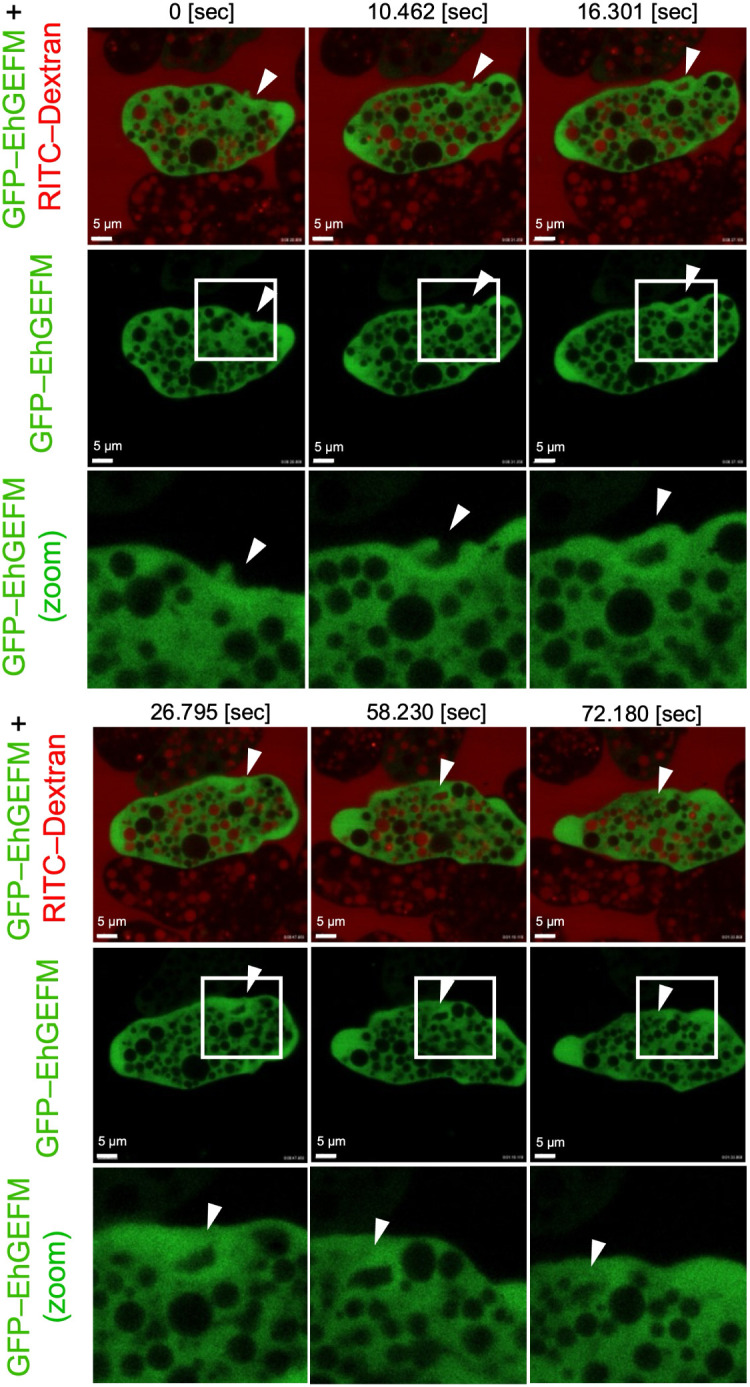
**Localization of GFP–EhGEFM during the initiation of macropinocytosis.** Montage of live imaging of GFP–EhGEFM-overexpressing trophozoites (green) that were incubated in RITC–dextran-containing medium (red). The white arrowheads show the site of macropinocytic cup formation and the resultant macropinosome. The regions enclosed by the white squares are displayed below (zoom). Images representative of multiple macropinocytosis events observed in one live-cell imaging. Scale bars: 5 μm.

**Fig. 7.**
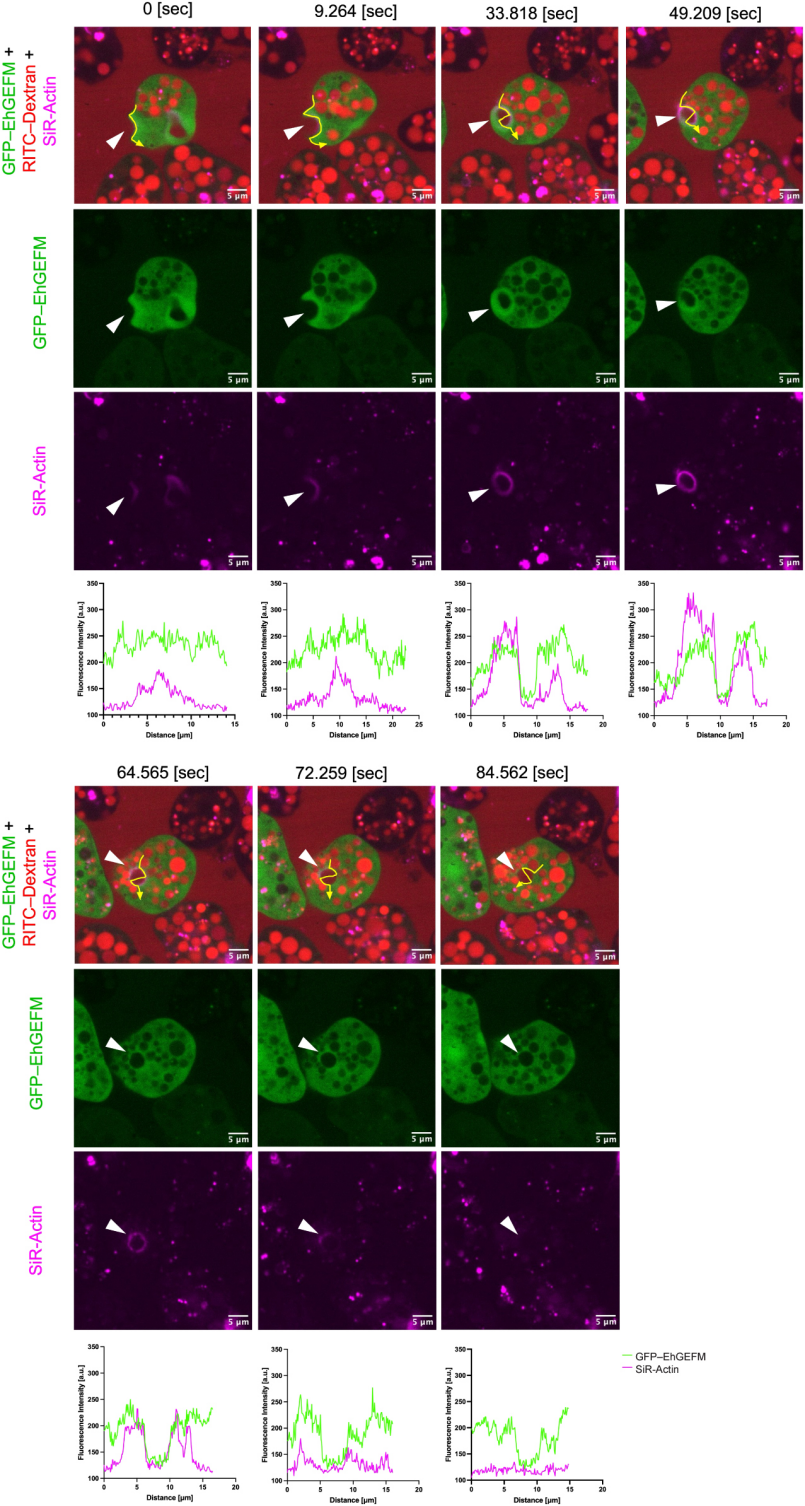
**Localization of GFP–EhGEFM and F-actin during macropinocytosis.** Montage of live imaging of GFP–EhGEFM-overexpressing trophozoites (green) that were incubated in RITC–dextran-containing medium (red). The magenta indicates SiR-Actin staining, which is an F-actin marker. The white arrowheads show the site of macropinocytic cup formation and the resultant macropinosome. The linescans along the yellow arrows, which demonstrate GFP–EhGEFM (light green) and SiR-Actin (magenta) dynamics at the macropinocytic cup and macropinosome formation, are displayed at the bottom. Images representative of multiple macropinocytosis events observed in one live-cell imaging. Scale bars: 5 μm.

To better understand how *EhgefM* gene silencing impairs directional cell migration while having no detectable effect on macropinocytosis, we examined the temporal dynamics of the subcellular localization of EhGEFM and EhRacM in *E. histolytica* trophozoites. We generated transformants expressing EhGEFM fused at the N-terminus to either an HA or GFP tag (HA–EhGEFM or GFP–EhGEFM, respectively) (Nakada-Tsukui et al., 2009; Somlata et al., 2017). Protein expression was confirmed by immunoblotting with anti-HA and anti-GFP antibodies. Both anti-HA and anti-GFP antibodies detected bands corresponding to the expected molecular mass of HA–EhGEFM (85.7 kDa for EhGEFM plus three 1.1 kDa HA tags, 89.0 kDa) and GFP–EhGEFM (85.7 kDa for EhGEFM plus 26.9 kDa for the GFP tag, 112.6 kDa) (Fig. 4A,C). An immunofluorescence assay (IFA) revealed that HA–EhGEFM predominantly localized in the cytosol, with notable enrichment at the cell periphery, particularly within pseudopods and macropinocytic cups (Fig. 4B). Occasional localization to the periphery of intracellular vesicles, presumably nascent macropinosomes, was also observed (Figs 4B and 5). These localization patterns were further confirmed by live-cell imaging of GFP–EhGEFM, which exhibited a diffuse and largely cytosolic pattern with enrichment at pseudopods and macropinocytic cups (Figs 4D, 6 and 7; Movies 3, 4, and 5). The discrepancy between HA- and GFP-tagged constructs likely reflects technical factors, such as tag size, expression level and detection sensitivity. Together, the data support a model in which EhGEFM is mainly cytosolic but can be transiently recruited to the cortex, including pseudopods and macropinocytic cups.

We further examined the cellular distribution of EhGEFM and EhRacM by IFA with anti-HA and anti-EhRacM antibodies. The anti-EhRacM antibody, commercially generated against recombinant EhRacM protein, was validated to detect both endogenous and exogenous (HA-fused) EhRacM by immunoblotting (Fig. S5A–D). The distribution of EhRacM in HA–EhRacM-overexpressing cells, as detected by anti-EhRacM antibody, overlapped with that detected using the anti-HA antibody on the intracellular vesicle surfaces, likely corresponding to macropinosomes (Fig. S5E). However, anti-EhRacM exhibited more cytosolic localization with notable enrichment at the cell periphery and pseudopods (Fig. S5E,F). In HA–EhGEFM-overexpressing trophozoites, EhRacM showed pronounced peripheral localization – particularly in pseudopods – and was occasionally enriched on intracellular vesicles, likely corresponding to nascent macropinosomes (Fig. 5).

### EhGEFM was recruited during macropinocytic cup formation and persists slightly beyond F-actin disassembly

Finally, to investigate the spatiotemporal dynamics of EhGEFM during macropinocytosis, we performed live-cell imaging of GFP–EhGEFM-overexpressing trophozoites in RITC–dextran-containing medium. GFP–EhGEFM accumulated at macropinocytic cups and on nascent macropinosomes, indicating its possible involvement in the early stages of macropinocytosis (Fig. 6; Movie 4).

Although endogenous EhRacM showed peripheral localization, including at pseudopods and nascent macropinosomes (Fig. 5), HA- and GFP-tagged EhRacM is mainly recruited to macropinosomes after the dissociation of the F-actin coat, and was absent during the initial cup formation stage (Shimoyama et al., 2024). Based on this, we next sought to infer the relative timing of EhGEFM and EhRacM recruitment. To this end, we performed live-cell imaging using SiR-Actin to monitor F-actin dynamics alongside GFP–EhGEFM. Note that compatibility of SiR-Actin staining with phalloidin labeling was previously confirmed (Shimoyama et al., 2024). We also verified that SiR-Actin staining under the conditions used here does not significantly affect macropinocytosis, phagocytosis or trogocytosis (Fig. S6).

Consistent with previous reports (Manich et al., 2018), F-actin was recruited to the forming macropinocytic cup and remained on the macropinosome for ∼1 min before dissociating. In contrast, GFP–EhGEFM was recruited to the macropinocytic cup and persisted on the macropinosome for ∼80 s, which was slightly longer than the F-actin envelope removal (Fig. 7; Movie 5). Taken together, these results indicate that EhGEFM is recruited during macropinocytic cup formation and remains associated with nascent macropinosomes slightly beyond F-actin disassembly. EhRacM, by contrast, accumulates on macropinosome surfaces after the dissociation of the F-actin coat under overexpression conditions, but its endogenous localization also includes the periphery of the cells, suggesting that EhGEFM and EhRacM might interact not only during macropinosome maturation but also at peripheral sites such as pseudopods and forming cups.

## DISCUSSION

### EhGEFM is a specific RhoGEF for EhRacM

EhGEFM was originally identified as a potential EhRacM interactor through interactome analysis of EhRacM (Shimoyama et al., 2024). In this study, we confirmed their interaction through co-IP (Table 1). Among several Rho small GTPases detected, including EhRacG and EhRacQ, EhRacM stood out for its consistent presence across all co-IP replicates and a higher QV ratio than the background, supporting its identity as the primary and specific substrate of EhGEFM (Table 1; Table S3).

*In vitro* RhoGEF exchange assays further demonstrated that EhGEFM selectively activates EhRacM (Fig. 1). Interestingly, only the truncated form of EhGEFM lacking the N-terminal Arm repeats (His–EhGEFMΔArm) exhibited catalytic activity, suggesting an autoinhibitory role for this domain. This contrasts with other EhRhoGEFs, such as EhGEF2, where Arm repeats are essential for its activity (González De la Rosa et al., 2007). Autoinhibition mechanisms have also been documented in other DH-domain-containing GEFs, including the proto-oncogene Vav (Aghazadeh et al., 2000) and Rac-specific GEF Asef (Mitin et al., 2007; Murayama et al., 2007). Furthermore, in some regulator of G-protein signaling (RGS)-domain-containing RhoGEFs, interaction with Gα12 or Gα13 subunits induces conformational changes that relieve autoinhibition by displacing the RGS-DH linker from the DH domain surface (Zheng et al., 2009). Although the precise mechanisms remain unclear, the Arm repeats in EhGEFM might serve a similar regulatory function, potentially mediating protein–protein interactions that alter the conformation of the DH–PH domain to facilitate interaction with EhRacM (Andrade et al., 2001). Further structural and biochemical studies are needed to validate this hypothesis.

The molecular basis for the substrate specificity of EhGEFM remains elusive. Despite ∼46% overall amino acid identity and the good conservation of the key residues in the switch regions between EhRacM and EhRho1B, EhRho1B was not efficiently activated by EhGEFM. AlphaFold2-based complex prediction did not explain this difference, as its plausible models for both EhRacM–EhGEFM and EhRho1B–EhGEFM revealed only equivalent residues in the canonical switch regions as the potential contact sites between EhRacM or EhRho1B and the DH domain of EhGEFM (EhRacM, Pro42, Thr43, Asp46 in switch I and Gly68, Phe72, Arg74 in switch II; EhRho1B, Pro51, Thr52, Glu55 in switch I and Gly77, Arg83, Leu87 in switch II) (see Fig. S7C,D for EhRacM–EhGEFM). However, comparison of residues corresponding to the human RhoA (HsRhoA)–Dbs complex (Snyder et al., 2002) (e.g. Arg5, Val43, Asp45, Glu54, Ala56 and Trp58) (Fig. S7B) identified that three of these residues (Val43, Asp45 and Ala56 in HsRhoA, corresponding to Leu49, Lys51 and Glu62 in EhRacM) are replaced with amino acids of different charge, polarity or size in EhRho1B (Ser58, Val60 and His71), potentially altering GEF-binding affinity.

### The EhGEFM–EhRacM axis governs directional migration but is dispensable for negative regulation of macropinocytosis

Silencing of *EhgefM* impaired directional migration in a manner similar to *EhracM* gene silencing (Fig. 3C) (Shimoyama et al., 2024), suggesting that EhGEFM functions upstream of EhRacM in trophozoite motility. Consistent with this hypothesis, EhGEFM localized predominantly to the cell periphery, including pseudopods, showing a similar distribution to endogenous EhRacM (Figs 4, 5; Fig. S5F and Movie 3). EhGEFM also interacted with actin-related proteins (Table S3). These observations support the hypothesis that EhGEFM facilitates directional persistence by locally activating EhRacM, thereby regulating actin dynamics to stabilize lamellipodia and maintain front–rear polarity (Iden and Collard, 2008; Krause and Gautreau, 2014).

Whereas EhRacM acts as a negative regulator of macropinocytosis as its gene silencing enhances fluid uptake (Shimoyama et al., 2024), silencing of *EhgefM* had no measurable effect on macropinocytosis. This result was unexpected, given that EhGEFM functions upstream of EhRacM in directional migration and is also transiently recruited to macropinocytic cups. Live-cell imaging revealed that GFP–EhGEFM is recruited to macropinocytic cups at an early stage and progressively dissociates once the actin coat is disassembled, preceding the major recruitment of EhRacM to mature macropinosomes (Shimoyama et al., 2024). These observations suggest that EhGEFM might interact with EhRacM at the periphery or early cup stage, but that this interaction is not essential for the overall progression of macropinocytosis. Thus, unlike its indispensable role in pseudopod-driven motility, EhGEFM appears dispensable for macropinocytosis, implying that other upstream regulators govern EhRacM activity during macropinosome maturation. However, we cannot exclude the possibility that residual EhGEFM protein persists in the silenced strain despite reduced mRNA levels, as protein abundance does not always directly correlate with transcript levels.

The idea that EhGEFM preferentially regulates directional migration is further supported by the phenotype of the HA–EhGEFMΔArm-overexpressing strain. Given that the Arm repeats appeared to exert an autoinhibitory effect in the *in vitro* GEF assay (Fig. 1A), deletion of this region was expected to relieve this inhibition and thereby enhance EhGEFM activity toward EhRacM. Indeed, the HA–EhGEFMΔArm-overexpressing strain tended to show a higher confinement ratio (Fig. 3E), indicative of more persistent and linear migration (Fig. S4D). This trend is consistent with the notion that increased EhGEFM–EhRacM signaling preferentially promotes directional migration. The relatively modest phenotype of the HA–EhGEFMΔArm-overexpressing strain compared with the robust activation observed *in vitro* further suggests that EhGEFM activity might be modulated by additional regulatory mechanisms *in vivo*.

Overexpression of HA–EhGEFMΔArm slightly increased macropinocytosis efficiency compared with what was seen for the mock control or HA–EhGEFM-overexpressing strains (Fig. S4B). This effect might be attributed to aberrant activation of EhRacM, potentially disrupting negative regulatory signals of EhRacM controlling macropinocytosis and resulting in a phenotype similar to that observed upon *EhracM* gene silencing. In addition, the slower growth of the HA–EhGEFMΔArm-overexpressing strain might also indicate that deregulated EhGEFM activity affects broader cellular processes. These points require further investigation.

### Temporal dynamics of EhGEFM during macropinocytosis maturation

Although we found that EhGEFM is dispensable for macropinocytosis, live-cell imaging using SiR-Actin and GFP–EhGEFM revealed that disassembly of the actin coat from newly formed macropinosomes precedes the release of EhGEFM. More precisely, F-actin dissociates ∼60 s after macropinosome closure, whereas EhGEFM dissociates ∼80 s post-formation (Fig. 7). This temporal order was unexpected, as Rho GTPases and their GEFs are typically associated with promoting actin polymerization and are presumed to disengage prior to, or concomitantly with, actin disassembly. These findings suggest that EhGEFM might not only function in actin polymerization at the periphery but might also transiently activate EhRacM or other small GTPases at nascent macropinosomes after actin disassembly. Such late-stage activation could serve roles unrelated to actin assembly, such as signaling or vesicle trafficking. Interestingly, Rac1 activation has also been observed on phagosomes undergoing actin disassembly (Hoppe and Swanson, 2004). Thus, a similar process might also underlie *E. histolytica* macropinocytosis.

### Potential upstream signals regulating the EhGEFM–EhRacM axis

Our co-IP results suggest a possible molecular context involving the EhGEFM–EhRacM axis. Among the 168 proteins identified in the HA–EhGEFM interactome, 123 were also found in the HA–EhRacM interactome (Table S3), strongly suggesting potential functional linkage or involvement in overlapping regulatory pathways. These included proteins related to ribosome biogenesis, proteasome function, and cytoskeletal regulation. Notably, the G protein α-subunit [EHI_140350, EhGα1 (Bosch et al., 2012)] was detected in both interactomes. In *E. histolytica*, EhGα1 has been shown to functionally resemble mammalian Gα12 and 13 in its ability to engage and activate an RGS-RhoGEF effector (Bosch et al., 2013), raising the possibility that it might serve as an upstream regulator of EhGEFM. Although EhGEFM lacks a canonical RGS domain, its Arm repeats might provide an alternative interaction surface, consistent with reports of the non-RGS RhoGEF proto-Dbl being activated by Gα13 in mammalian cells. Further studies are warranted to investigate whether EhGα1 directly regulates EhGEFM activity.

The presence of a Rho GDP-dissociation inhibitor (RhoGDI, EHI_147570) suggests that the EhGEFM–EhRacM axis might also be modulated at the level of Rac sequestration in the cytosol (Mosaddeghzadeh and Ahmadian, 2021). In addition, the identification of several AP-related proteins (EHI_083430, EHI_013040 and EHI_164810) in both the EhRacM and EhGEFM interactomes further supports a functional link between the EhGEFM–EhRacM axis and endosomal trafficking (Field and Dacks, 2009; Perdomo et al., 2015). Notably, EHI_083430 and EHI_164810 are annotated as subunits of the AP-3 complex, which in mammalian cells is involved in transporting membrane proteins and cargo from the trans-Golgi network (TGN) and/or endosomes to lysosomes or lysosome-related organelles. The identification of cytoskeletal proteins, including cortexillin (EHI_191900), among the shared interactors further supports a role of the EhGEFM–EhRacM axis in actin cytoskeleton organization.

In summary, this study provides the first identification and functional characterization of EhGEFM as a RhoGEF that specifically regulates EhRacM and primarily contributes to directional cell migration (Fig. S8), while being dispensable for EhRacM-mediated negative regulation of macropinocytosis. The localization of EhGEFM and its timing of dissociation from macropinosomes suggest a possible role during early cup formation and nascent macropinosome dynamics. Considering its structure and potential interactors, EhGEFM might act as a non-canonical G-protein-responsive RhoGEF that links upstream signaling to cytoskeletal dynamics and vesicle trafficking. Further investigation into the EhGEFM–EhRacM axis and its upstream regulators will advance our understanding of cellular behavior and pathogenesis in *E. histolytica*.

## MATERIALS AND METHODS

### Organisms, cultivation and reagents

Trophozoites of *E. histolytica* clonal strains HM-1:IMSS cl6 and G3 strain were obtained from laboratory stocks. Cells were not recently authenticated, and cultures were not tested for mycoplasma contamination but were routinely monitored for microbial contamination. Trophozoites were cultured axenically in 6 ml screw-capped Pyrex glass tubes in Diamond's BI-S-33 (BIS) medium at 35.5°C as previously described (Bracha et al., 2006; Diamond et al., 1978, 1972). Phosphate-buffered saline (PBS) and Rhodamine B isothiocyanate–Dextran (RITC–Dextran) were purchased from Sigma-Aldrich (Missouri, USA). Anti-GFP monoclonal mouse antibody (clones 7.1 and 13.1, cat. no. 11814460001) was purchased from Merck (Mannheim, Germany). The anti-HA 16B12 monoclonal mouse antibody (cat. no. 901501) was purchased from Biolegend (San Diego, USA). Lipofectamine, PLUS reagent and geneticin (G418) were purchased from Invitrogen. CellTracker™ Deep Red was purchased from Thermo Fisher Scientific (Massachusetts, USA). Unless otherwise mentioned, restriction enzymes and DNA-modifying enzymes were purchased from New England Biolabs (Massachusetts, USA). Other common reagents were from Fujifilm Wako Pure Chemical (Osaka, Japan) unless otherwise stated. Plasmids and other reagents generated in this study are available from the corresponding author upon reasonable request, subject to institutional guidelines.

### Establishment of *E. histolytica* transformants

Transformants of *E. histolytica* were generated as described previously (Shimoyama et al., 2024), with modifications for *EhgefM*. Briefly, for antisense small RNA-mediated transcriptional silencing, around a 420 bp fragment of the protein-coding region of the gene (Table S1) was amplified by PCR from cDNA with sense and antisense oligonucleotides containing StuI and SacI restriction sites (Table S2) and cloned into psAP2-Gunma plasmid (Mi-ichi et al., 2011) to synthesize a gene silencing plasmid designated as psAP2-EhGEFM. For overexpression constructs, either the full-length of the protein-coding region of *EhgefM* (Table S1) was amplified by PCR from cDNA with sense and antisense oligonucleotides containing SmaI and XhoI restriction sites (Table S2) and cloned into pEhEx-HA (Nakada-Tsukui et al., 2012) or pEhEx-GFP (Yousuf et al., 2010) vectors to produce pEhExHA-EhGEFM, or pEhExGFP-EhGEFM. For the overexpression construct of EhGEFMΔArm, inverse PCR was used to amplify the region containing DH and PH domains (aa 391–738) while excluding the remaining regions of EhGEFM, using pEhExHA-EhGEFM as a template with sense and antisense oligonucleotides. The PCR product was assembled and circularized using the Mighty Cloning Kit (Takara, Tokyo, Japan) according to the manufacturer's instructions. pEhExHA-EhGEFM, pEhExHA-EhGEFMΔArm and pEhExGFP-EhGEFM were introduced into the trophozoites of *E. histolytica* HM-1:IMSS cl6 strain, whereas psAP2-EhGEFM was introduced into G3 strain by lipofection as described previously (Nozaki et al., 1999). Transformants were initially selected in the presence of 1 μg ml^−1^ G418 until the drug concentration was gradually increased to 10 μg/ml, and all transformants were maintained at 10 μg ml^−1^ G418 in BIS medium.

### Reverse transcriptase PCR

Reverse transcriptase PCR was performed to check mRNA levels of *EhgefM* in the psAP2-EhGEFM strain and the mock control strain (psAP2), as described previously (Shimoyama et al., 2024). ExTaq PCR system was used to amplify DNA from the cDNA template using the primer pairs listed in Table S2, and the PCR products obtained were resolved by agarose gel electrophoresis.

### qRT-PCR

The relative mRNA levels of *EhgefM* and *RNA polymerase II* gene (EHI_056690), as an internal standard, were measured by qRT-PCR as described previously (Shimoyama et al., 2024). The PCR reaction was prepared using Fast SYBR Master Mix (Applied Biosystems, California, USA) with cDNA and the primer set shown in Table S2. The mRNA expression level of the *EhgefM* gene in the transformants was presented relative to that in the control transfected with psAP2.

### Macropinocytosis efficiency evaluation by flow cytometer

Macropinocytosis was quantified as described previously (Shimoyama et al., 2024). Trophozoites were incubated in BIS medium containing 2 mg ml^−1^ RITC–dextran (70 kDa) for the indicated time, then washed three times with cold PBS. Fluorescence was measured using a BD Accuri™ C6 Plus flow cytometer (Becton Dickinson, USA), and data were analyzed with FlowJo software. Geometric mean fluorescence intensity in the PE-A channel, after subtraction of background signal from unlabeled parasites, was normalized to the psAP2 mock control strain at the longest time point of each assay. Statistical significance was assessed using an unpaired two-tailed *t*-test across three independent experiments.

### Motility assay

Trophozoites of the *EhgefM* gene-silenced strain and the psAP2 mock control strain were stained in Opti-MEM (Thermo Fisher Scientific) containing 1 mg ml^−1^ of ascorbic acid and 5 mg ml^−1^ of L-cysteine, as well as 10 μM of CellTracker™ Deep Red Dye for 40 min at 35.5°C. Cells were then washed, resuspended in BIS medium, and imaged using a CQ1 confocal quantitative image cytometer (Yokogawa Electric, Japan) at 37°C with 1-s intervals for 100 frames. Cell motility parameters were quantified using CellPathfinder software (Yokogawa Electric) as described previously (Shimoyama et al., 2024).

### Immunoblot analysis

Immunoblotting was performed as described previously (Shimoyama et al., 2024). Briefly, trophozoites of amoeba transformants expressing HA–EhGEFM, HA–EhGEFMΔArm or GFP–EhRacM were lysed, and ∼30 μg of the total cell lysates were separated on 12% SDS-PAGE and subsequently electrotransferred onto nitrocellulose membranes. The membranes were probed with anti-HA 16B12 monoclonal mouse antibody (1:1000), anti-GFP mouse monoclonal antibody (1:100), and anti-cysteine synthase 1 (CS1) rabbit polyclonal antisera (Nozaki et al., 1998) (1:1000), followed by horseradish peroxidase-conjugated (HRP) anti-mouse-IgG (Invitrogen) (1:1000) or anti-rabbit-IgG antisera (Thermo Fisher Scientific) (1:10,000) and visualized with a chemiluminescence HRP Substrate system (Merck) using ChemiDoc Imaging System (Biorad, California, USA). Source data for immunoblots, including uncropped images, are shown in Fig. S9A,B.

### IFA

The IFA was performed as described previously (Shimoyama et al., 2024). Briefly, trophozoites of the transformant strain expressing HA–EhGEFM were fixed with PBS containing 3.7% paraformaldehyde (Kanto Chemical Co., Inc., Tokyo, Japan), permeabilized with 0.2% Saponin (Sigma-Aldrich) in 1% bovine serum albumin (BSA; Sigma-Aldrich), and stained with anti-HA mouse monoclonal antibody (1:1000) followed by Alexa Fluor 488-conjugated goat anti-mouse IgG (H+L), Superclonal™ recombinant secondary antibody (1:1000) (Thermo Fisher Scientific). The samples were mounted in the mounting medium [1 mg/ml p-phenylenediamine (Sigma-Aldrich) in a mixture of 90% glycerol and PBS] and imaged by FV3000 Confocal Microscope System (Olympus) using a 60× oil immersion objective with default settings and analyzed by Fiji-ImageJ software (Schindelin et al., 2012).

### EhRacM and HA double staining IFA

Double immunofluorescence staining was performed essentially as described above, with the addition of anti-EhRacM antibody. Briefly, trophozoites of the transformant strain expressing HA–EhGEFM were fixed with PBS containing 3.7% paraformaldehyde, permeabilized with 0.2% Saponin in 1% BSA, and incubated with an anti-HA mouse monoclonal antibody (1:1000) and an anti-EhRacM rabbit polyclonal antibody (1:100). The sample reacted with Alexa Fluor 488-conjugated goat anti-mouse IgG (H+L), Superclonal™ recombinant secondary antibody (1:1000) and Alexa Fluor-568 conjugated goat anti-rabbit IgG (H+L) cross-adsorbed secondary antibody (1:1000) (Thermo Fisher Scientific). The samples were mounted and imaged by FV3000 Confocal Microscope System and analyzed by Fiji-ImageJ software.

### Recombinant proteins

To produce recombinant full-length and truncated EhGEFM proteins, the full-length sequence or a DH–PH-containing fragment (aa 391–738; codon-optimized) from cDNA or a commercially synthesized plasmid were used (Table S2). The products were subcloned into pCold I vector (Takara) and expressed as Histidine (His) tag-fused recombinant protein (His–EhGEFM or His-EhGEFMΔArm, respectively). To produce GST-fused EhRho and EhRac, the open reading frames of *Ehrho* and *Ehrac* genes {EHI_029020 [*Ehrho1B* (Chung et al., 2010)], EHI_129750 [*EhracG* (Chung et al., 2010)], EHI_012240 [*EhracD1* (Chung et al., 2010)], EHI_197840 [*EhracA2* (Chung et al., 2010)] or EHI_135450 [*EhracM* (Chung et al., 2010)]} (Table S1) were PCR-amplified (Table S2) and subcloned into pCold-GST vector, which contains a GST tag following its His_6_ tag (Takara). PCR was performed with Tks Gflex™ DNA Polymerase (Takara) with the following parameters: initial denaturation at 94°C for 1 min; then 35 cycles at 98°C for 10 s, 58°C or 60°C for 15 s, and 68°C for 30 s kb^−1^; and a final extension at 68°C for 1 min.

The plasmids were transformed separately into *Escherichia coli* BL21 (DE3) Singles™ Competent cells (Merck) for effective protein expression. Each clone of the plasmids was grown at 37°C in Luria–Bertani broth, Miller (LB) medium (Nacalai Tesque, Kyoto, Japan), containing 100 mg l^−1^ ampicillin under shaking overnight, and these cultures were diluted to an optical density at 600 nm (OD600)=0.02 in 1 l fresh LB medium and allowed to grow at 37°C to middle log phase (OD600 between 0.4 and 0.6). The proteins were expressed by cold-shock treatment followed by induction with isopropyl-β-D-thiogalactoside (IPTG) (Nacalai Tesque); namely, the medium was cooled in ice-water mixture to drop the medium temperature, and kept in the ice bath for an additional 20 min. After cold-shock treatment, expressions of each protein were induced with either 0.5 mM IPTG [GST–EhRacG, GST–EhRacD1, GST–EhRacA2, GST–EhRacM and His–EhGEFM)] or 0.3 mM IPTG [GST–EhRho1B and His–EhGEFMΔArm] for 46 h at 10°C. The cells were harvested by centrifugation at 7500 ***g*** for 10 min at 4°C, and the centrifuged pellets were then washed with Tris-buffered saline (10 mM Tris-HCl pH 7.6 and 150 mM NaCl).

The pellets were resuspended in ice-chilled lysis buffer [50 mM Tris-HCl pH 7.6, 250 mM NaCl, 10% sucrose, containing 0.5 mg ml^−1^ E-64, cOmplete™, Mini, EDTA-free protease inhibitor (Roche, Basel, Switzerland), 1 μM PMSF, and 0.5 mg ml^−1^ lysozyme, from egg white]. Then, pellets were kept on ice for 10 min and lysed by French Press (Ohtake, Tokyo, Japan). Then, 0.1% Triton-X 100 was added to the lysates and kept on ice for 10 min. The lysates were centrifuged at 16,000 ***g*** for 30 min at 4°C, and the supernatants were collected and filtered for further purification. For His–EhGEFM and His–EhGEFMΔArm, after washing with PBS three times, TALON^®^ Metal Affinity Resin (Takara) was mixed with the supernatant. For GST-tag fused EhRho and EhRacs, glutathione Sepharose™ 4 Fast Flow Resin (Cytiva, Massachusetts, USA) was mixed with the supernatant. Then, the mixture was incubated at 4°C with rotation overnight. The mixtures of His–EhGEFM and His–EhGEFMΔArm were then loaded to Econo-Pac^®^ Disposable Chromatography Columns (Bio-Rad, California, USA), and the resin–supernatant reaction was subsequently washed with wash buffer (50 mM Tris-HCl pH 7.6 and 2 M KCl), and eluted with 10 mM, 30 mM, 50 mM, 100 mM, 200 mM, 300 mM and 750 mM imidazole. The mixtures of GST-tag fused proteins were loaded to Econo-Pac^®^ Disposable Chromatography Columns (Bio-Rad), and the resin–supernatant reaction was subsequently washed with wash buffer [5 mM 2-mercaptoethanol (Nacalai Tesque) in PBS] three times, and eluted with elution buffer [50 mM Tris-HCl and 15 mM reduced L-glutathione (Sigma), pH 8.0].

The GST tag of EhRacM was further cleaved by HRV 3C Protease (Takara) at 4°C with rotation overnight after changing the buffer from elution buffer to HRV 3C cleavage buffer (50 mM Tris-HCl pH 7.6 and 150 mM NaCl) using Amicon Ultra-4 15K tubes (Merck). The HRV 3C Protease and uncleaved GST–EhRacM were removed by rotating with TALON^®^ Metal Affinity Resin (Takara) at 4°C for 1.5 h, as they contain His tag.

### RhoGEF exchange assay

After the elution of His- or GST-tag-fused proteins, the elution buffers were changed to Exchange Buffer (20 mM Tris-HCl pH 7.6, 50 mM NaCl, and 10 mM MgCl_2_) using Amicon Ultra tubes (Merck). Then, the GEF activities of His–EhGEFM, His–EhGEFMΔArm and hDbs–His (Cytoskeleton, Inc., Colorado, USA) on GST-tagged EhRhos or EhRacs were measured using the RhoGEF Exchange Assay Biochem Kit (Cytoskeleton), following the manufacturer's protocol. 3.0×10^−12^ mol of GST–EhRhos or EhRacs were reacted with 7.5×10^−12^ mol of His–EhGEFM, His–EhGEFMΔArm or hDbs–His (total reaction volume was 15 μl), unless specific concentrations are mentioned, in the 384-well black round-bottom plate (Corning, New York, USA). The fluorescence was measured by a plate reader, setting the excitation filter wavelength at 485 nm and the emission filter wavelength at 515 nm.

The catalytic efficiency of EhGEFM for EhRho1B, EhRacD1 and EhRacM was determined using the method described previously (Apte et al., 2022). First, observed pseudo-first-order exchange rate constants (*K*_obs_) were obtained by a nonlinear least square fit of data at each concentration of EhGEFM to an exponential equation:

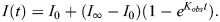
Here, *I*(*t*) is the intensity at time *t*, *I*_0_ is the initial intensity and *I*_∞_ is the final intensity. Then, the catalytic efficiency was estimated from the slope of a linear least square fit of the *K*_obs_ values across EhGEFM concentration:

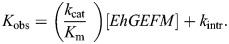
where *k*_intr_ is the intrinsic nucleotide exchange rate of each EhRho or EhRac substrate in the abscence of EhGEFM, *K_m_* is the Michaelis constant for the EhRho/EhRac substrate, and 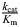 reflects catalytic efficiency of EhGEFM-mediated nucleotide exchange at low substrate concentrations.

### Production of EhRacM antibody

Anti-EhRacM antiserum was raised against recombinant EhRacM in rabbits commercially (Eurofinsgenomics, Tokyo, Japan). Source data for antibody validation by immunoblot are provided in Fig. S9C.

### Live-cell imaging and macropinocytosis imaging

As previously described (Shimoyama et al., 2024), trophozoites of the transformant strain expressing GFP–EhGEFM were cultured on a glass-bottom dish (MatTek Corporation, Massachusetts, USA) in filtered BIS medium at 35.5°C. For macropinocytosis live imaging, the BIS medium was replaced with BIS medium containing RITC–dextran prior to imaging. Live images were captured by Andor Dragonfly 200 Spinning Disk Confocal Microscope System (Oxford Instruments, Abingdon, UK) using a 60× oil immersion objective and analyzed by IMARIS software (Oxford Instruments).

### Macropinocytosis live imaging stained by SiR-Actin

Trophozoites of the transformant strain expressing GFP–EhGEFM were suspended with 1 ml of filtered BIS medium containing 10 μM SiR-Actin (Cytoskeleton, Inc.) and 10 μM Verapamil (Cytoskeleton, Inc.) and cultured on a 35 mm glass-bottom dish for 1 h at 37°C, as described previously (Shimoyama et al., 2024). Live imaging was performed as described above.

### Cross-linking and co-immunoprecipitation of HA-tagged EhGEFM

The cross-linking and co-immunoprecipitation (co-IP) assay was performed as described previously (Shimoyama et al., 2024). In brief, trophozoites of the amoeba transformants overexpressing HA–EhGEFM and pEhEx-HA (mock control) were cross-linked with 8 mg ml^−1^ dithiobis (succinimidyl propionate; DSP) solution (Thermo Fisher Scientific), lysed with 1 ml of lysis buffer (50 mM Tris-HCl pH 7.6, 150 mM NaCl, containing 1% Triton X-100, 0.05 mg/ml E-64, and cOmplete™, Mini, EDTA-free protease inhibitor), and incubated with 50 µl of monoclonal anti-HA-agarose antibody produced in mouse, clone HA-7, purified immunoglobulin conjugated to agarose beads (Sigma-Aldrich). HA-tagged protein complexes were eluted with Influenza Hemagglutinin (HA)-peptide (Sigma-Aldrich) and submitted to the Mass Spectrometry and Proteomics Core Facility, Johns Hopkins University School of Medicine, Baltimore, MD, USA, for mass spectrometry analysis.

### Mass spectrometry

Mass spectrometry sample preparation and analysis were performed as described previously (Shimoyama et al., 2024). The mass spectrometry proteomics data have been deposited to PRIDE (Perez-Riverol et al., 2022) partner repository with the dataset identifier PXD068844. The Gene Ontology (GO) analysis was conducted by PANTHER Overrepresentation Test (Released 20240807) with Fisher's Exact test, based on the GO Ontology database doi:10.5281/zenodo.7942786. The plots were mapped by the ggplot2 R package (3.5.1).

### Graphs and statistical analyses

Line plots and bar graphs in this paper were generated in GraphPad Prism 9.4.1. All the analyses were done in GraphPad Prism version 9.4.1. Detailed statistical methods are described in each figure.

Sample sizes were chosen based on previous studies in the field and were not determined by formal power analysis. No samples were excluded from the analysis, except in motility assays, where cells with a total distance traveled of <50 µm were excluded. Data were assumed to follow a normal distribution; however, this was not formally tested.

### Multiple sequence alignment of RhoGEF

Multiple sequence alignment was conducted by Clustal Omega (Madeira et al., 2024). The output was visualized by Jalview software (2.11.3.3) (Waterhouse et al., 2009).

### Protein 3D structures comparison

All predicted protein structures were obtained from the AlphaFold Protein Structure Database (https://alphafold.ebi.ac.uk/). Structural comparisons between proteins were performed using the align function in the PyMOL Molecular Graphics System (Version 3.0; Schrödinger, LLC).

### Generative AI tools usage

Proofreading and language editing assistance were provided using ChatGPT-5.5 Thinking (OpenAI) to improve the clarity and readability of the manuscript. After using these services, the authors reviewed and edited the content as needed and take full responsibility for the content of the publication.

## Supplementary Material



10.1242/joces.264490_sup1 Supplementary information

Table S1. Amino acid sequences of EhGEFM and EhRho/Rac proteins used in this study This table lists the amino acid sequences and annotation names of EhGEFM and EhRho/Rac proteins used in this study. The region of the EhGEFM sequence covered by the EhGEFMΔArm construct is highlighted in red.

Table S2. List of primers used in this study Restriction sites are marked by bold letters. S stands for sense strand, whereas AS stands for antisense strand.

Table S3. List of hits identified by HA–EhGEFM co-IP This table lists the hits identified in HA–EhGEFM co-IP, where the mean quantitative value (QV) from two replicates of the co-IP experiment is greater than that of the mock strain. The columns "HA–EhGEFM_1", "mock_1", "HA–EhGEFM_2", and "mock_2" represent the QV from the first and second co-IP of the HA–EhGEFM-overexpressing strain co-IP, and the corresponding QV for the mock strain, respectively. The "Average EhGEFM QV" column indicates the average QV from the two co-IPs of the HA–EhGEFM-overexpressing strain, while the "Average mock QV" column shows the mean QV from the mock strain. The "Average HA–EhGEFM QV/mock QV" column represents the ratio of the average HA–EhGEFM QV to the average mock QV. Note that hits from the HA–EhGEFM-overexpressing strain with QVs less than 1/100 of the bait protein (HA–EhGEFM) were excluded from this table. The hits are ordered by the HA–EhGEFM QV/mock QV ratio. Proteins related to Rho signaling are highlighted in red, those related to the actin cytoskeleton in coral pink, those related to the proteasome system in cream yellow, those associated with G proteins in yellow, and ribosome-related proteins in light green. The "Occurrences" column indicates how many times each hit was detected in previous HA–EhRacM co-IP experiments (n=3), where hits were defined by their QV being higher in the HA–EhRacM-overexpressing strain compared to the mock strain (Shimoyama et al., 2024).
