# Supplementary material for: A RhoGEF activates RacM to selectively promote motility without affecting macropinocytosis in Entamoeba histolytica
Source: J Cell Sci. 2026 Jun 16;139(11):jcs264490. doi: 10.1242/jcs.264490 (PMC13327543; doi:10.1242/jcs.264490)
Supplement: Supplementary information [file joces-139-264490-s1.pdf]

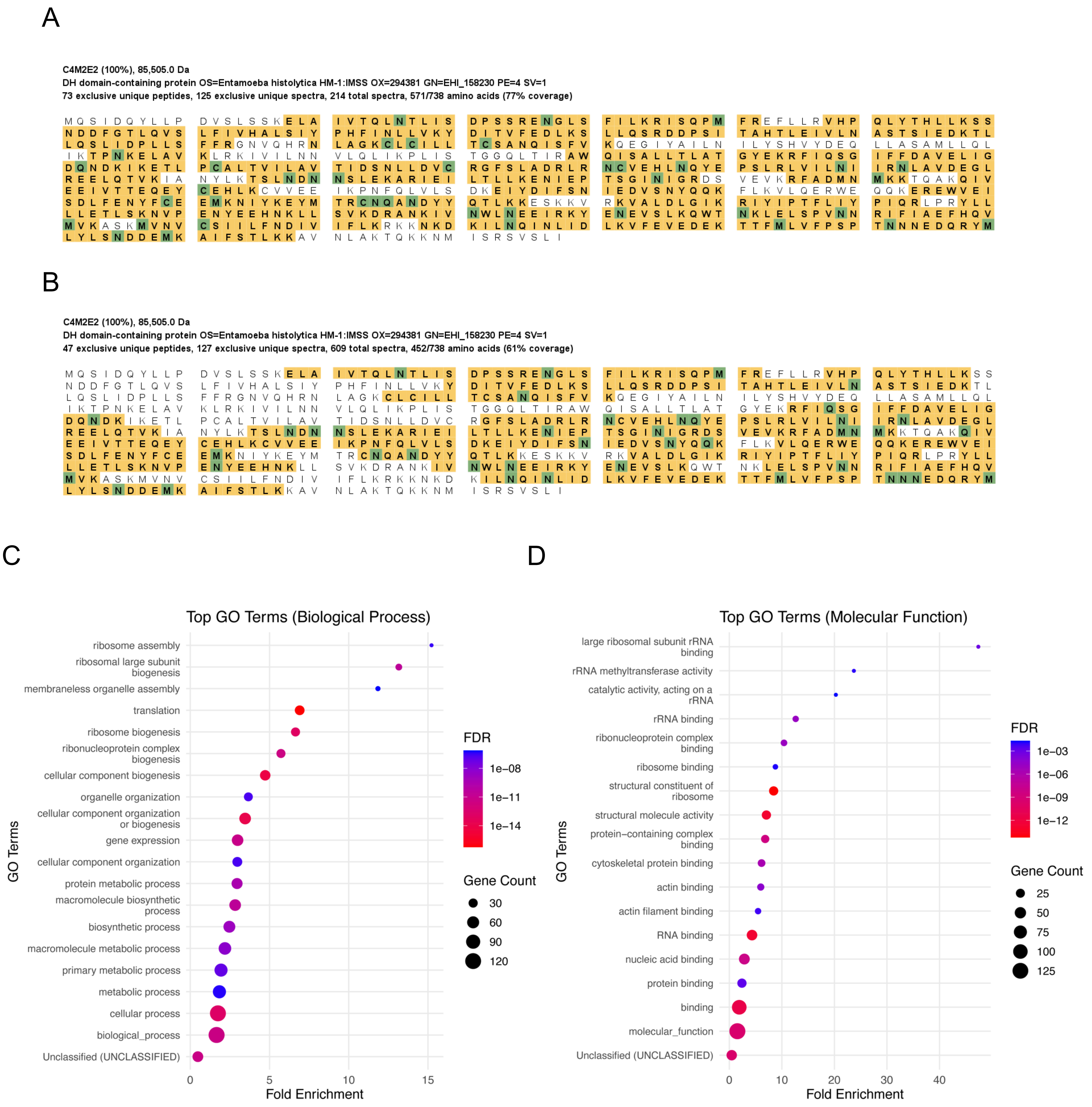

Fig. S1. Mass spectrometry identification of EhGEFM peptides and GO enrichment analysis of candidate binding proteins from HA–EhGEFM co-IP.

(A) and **(B)** show mass spectrometry identification of EhGEFM (C4M2E2) peptides; from the first co-IP sample **(A)**, and the second co-IP sample **(B)**, respectively. Yellow highlights indicate the detected peptides, with post-translational modifications (green), such as oxidation, deamidation, or carbamidomethylation. **(C)** and **(D)** show the results of PANTHER GO enrichment analysis of 168 hit proteins obtained from HA–EhGEFM co-IP. Proteins were classified by biological process (BP) **(C)** and molecular function (MF) **(D)**. GO terms are shown in ascending order of fold enrichment. Dot size indicates gene count, whereas color indicates FDR. The x-axis indicates fold change.

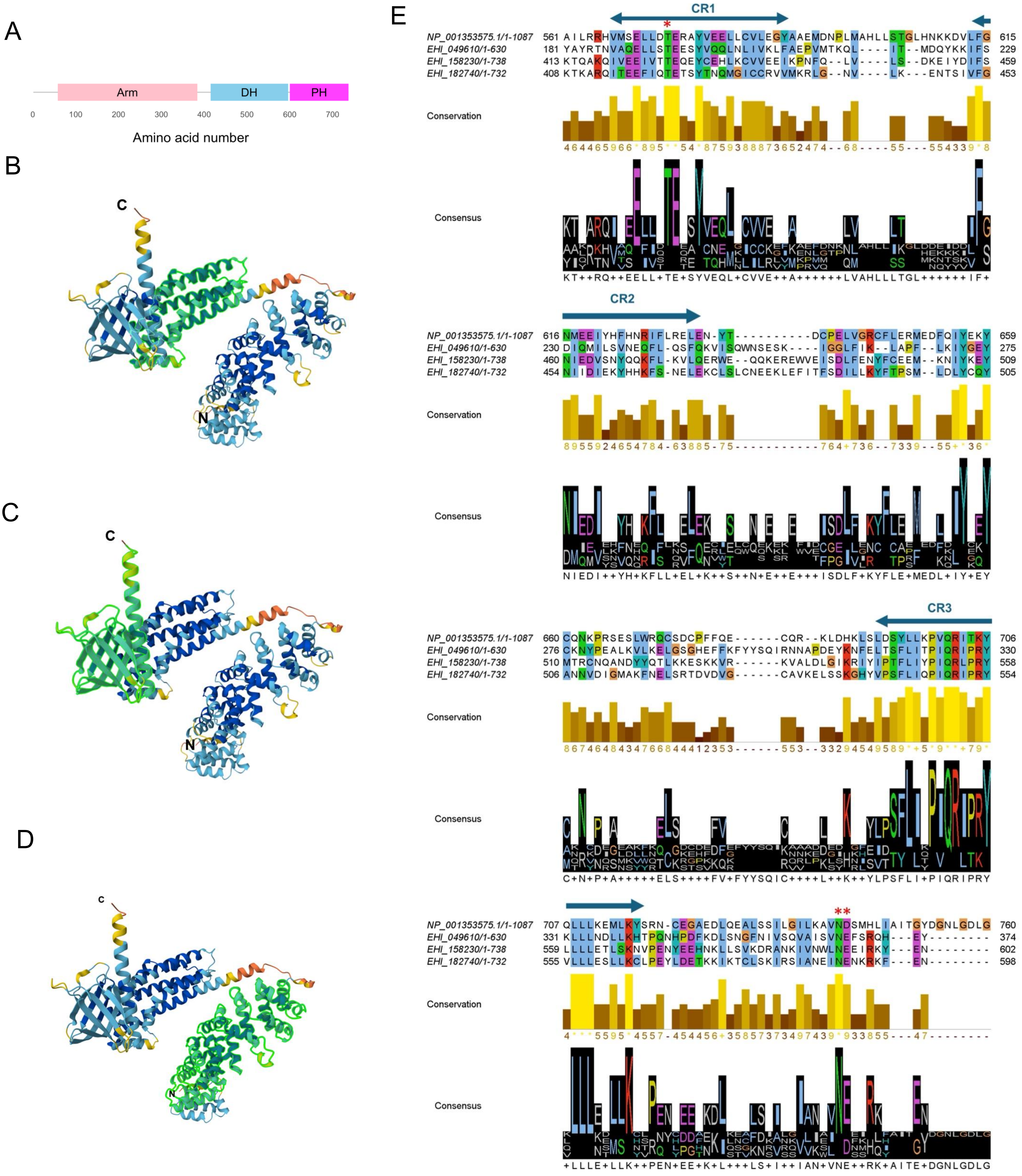

Journal of Cell Science • Supplementary information

F

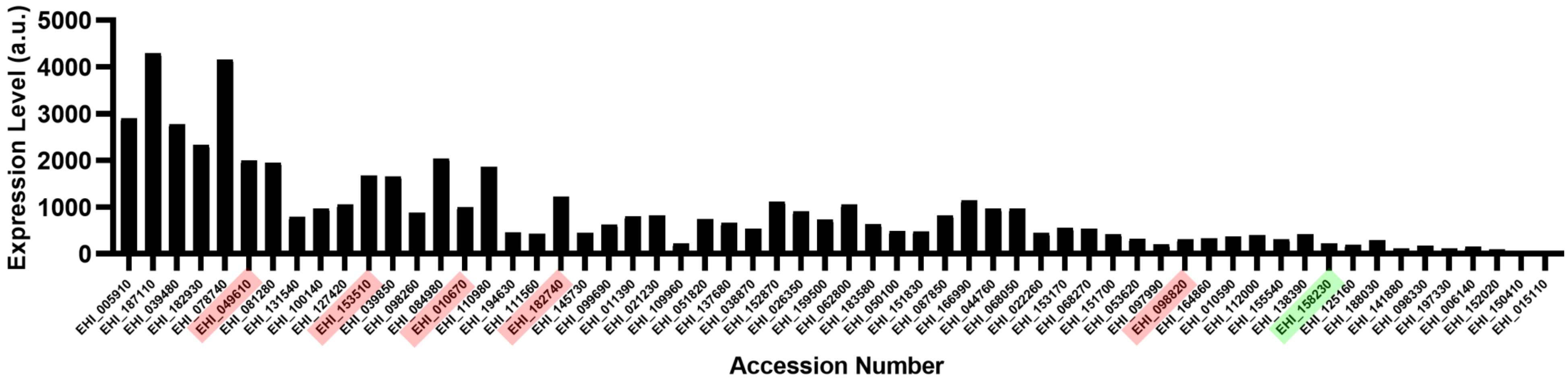

Fig. S2. Structural and sequence analysis of EhGEFM and its expression level. (A) Schematic representation of the domain structure of EhGEFM. Armadillo-like helical domain: aa 58–384, Dbl homology (DH) domain: aa 415–596, PH-like domain superfamily: aa 600–737. **(B)-(D)** Protein structure prediction by AlphaFold 2. In each panel, the indicated domain is highlighted in green: DH domain in (B), the PH domain in (C), and the Arm repeats in (D). N and C indicate the N-terminus and C-terminus of the EhGEFM protein. **(E)** Conservation of the DH domain among human and *E. histolytica* RhoGEFs. Multiple Sequence Alignment (MSA) of HsDbs (NP_001353575), EhGEF1 (EHI_049610), EhGEFM (EHI_158230), and EhGEF2 (EHI_182740) by Clustal Omega. Three conserved regions (CR1–CR3) are shown by double-headed arrows at the top. The key residues directly involved in the catalytic activity of DH RhoGEFs [threonine 426 (T426) and asparagine–glutamate pair at positions 594–595 (N594–E595)] are highlighted by red asterisks. Amino acid conservation ratios and consensus residues are shown in the middle and lower rows, respectively. **(F)** Expression level of RhoGEF measured by RNA-seq. Pink-colored ones are previously studied RhoGEF, EHI_049610 (EhGEF1), EHI_153510 (EhFP4), EHI_010670 (RGS domain-containing RhoGEF), EHI_182740 (EhGEF2), and EHI_098820 (EhGEF3), respectively, whereas green indicates EhGEFM. Expression levels were calculated from two technical replicates of RNA-seq analysis in HM-1:IMSS cl-6 strain (Tsukui, data not shown).

Journal of Cell Science • Supplementary information

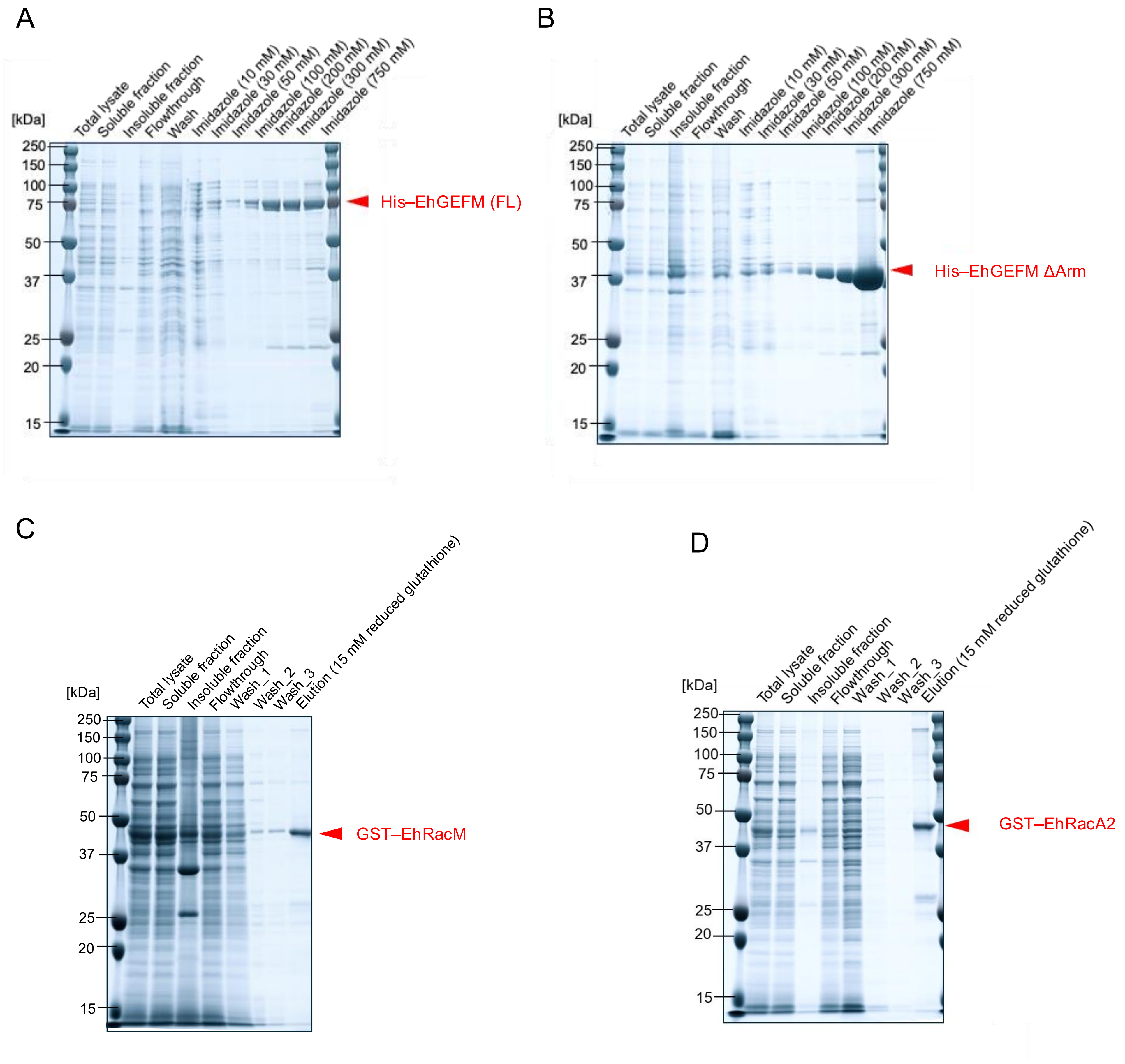
A
[kDa]
Total lysate
Soluble fraction
Insoluble fraction
Flowthrough
Wash
Imidazole (10 mM)
Imidazole (30 mM)
Imidazole (50 mM)
Imidazole (100 mM)
Imidazole (200 mM)
Imidazole (300 mM)
Imidazole (750 mM)
250
150
100
75
50
37
25
20
15
His–EhGEFM (FL)
B
[kDa]
Total lysate
Soluble fraction
Insoluble fraction
Flowthrough
Wash
Imidazole (10 mM)
Imidazole (30 mM)
Imidazole (50 mM)
Imidazole (100 mM)
Imidazole (200 mM)
Imidazole (300 mM)
Imidazole (750 mM)
250
150
100
75
50
37
25
20
15
His–EhGEFM ΔArm
C
[kDa]
Total lysate
Soluble fraction
Insoluble fraction
Flowthrough
Wash_1
Wash_2
Wash_3
Elution (15 mM reduced glutathione)
250
150
100
75
50
37
25
20
15
GST–EhRacM
D
[kDa]
Total lysate
Soluble fraction
Insoluble fraction
Flowthrough
Wash_1
Wash_2
Wash_3
Elution (15 mM reduced glutathione)
250
150
100
75
50
37
25
20
15
GST–EhRacA2

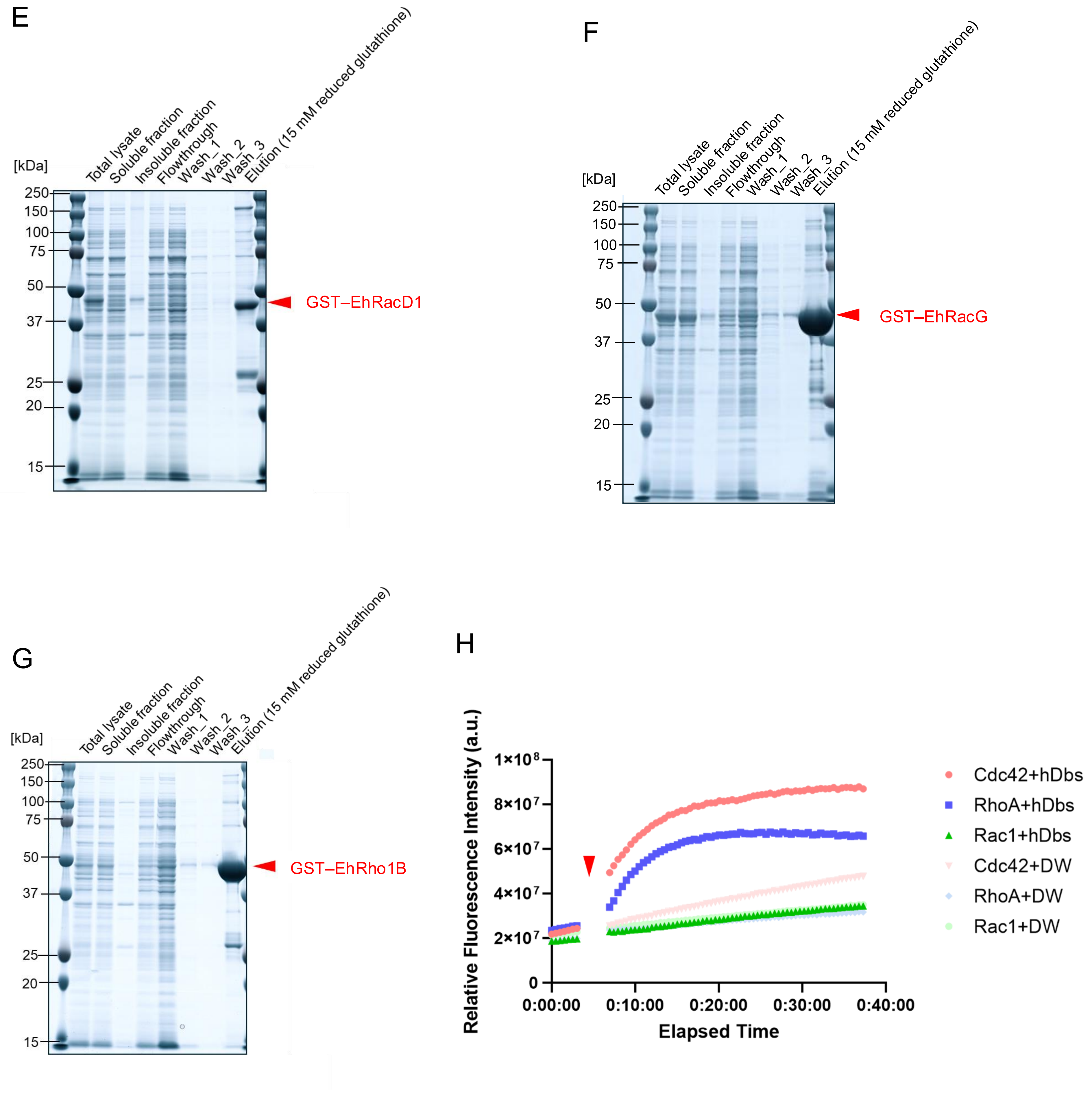

Fig. S3. Protein purification of recombinant proteins and validation of the RhoGEF exchange assay.
(A) - (G) Protein purification of recombinant EhGEFM and EhRho/EhRacs. Gel images show CBB staining after SDS-PAGE. **(A)** His–EhGEFM (FL). Predicted size: 88.4 kDa. **(B)** His–EhGEFMΔArm. Predicted size: 44.9 kDa. **(C)** GST–EhRacM. Predicted size: 51.1 kDa **(D)** GST–EhRacA2. Predicted size: 50.2 kDa **(E)** GST–EhRacD1. Predicted size: 51.0 kDa **(F)** GST–EhRacG. Predicted size: 50.8 kDa **(G)** GST–EhRho1. Predicted size: 52.4 kDa **(H)** Validation of the RhoGEF Assay. Relative fluorescence intensity was measured using a plate reader. After seven initial readings, either hDbs–His or distilled water (DW) was added (indicated by the red triangle) to the respective Rho small GTPase (human Cdc42–His, RhoA–His, or Rac1–His), and readings were resumed. A steeper fluorescence intensity curve indicates more efficient RhoGEF-mediated nucleotide exchange activity. Note that hDbs is known to be an efficient GEF for RhoA and Cdc42 GTPases but exhibits weaker GEF activity toward Rac1.

Journal of Cell Science • Supplementary information

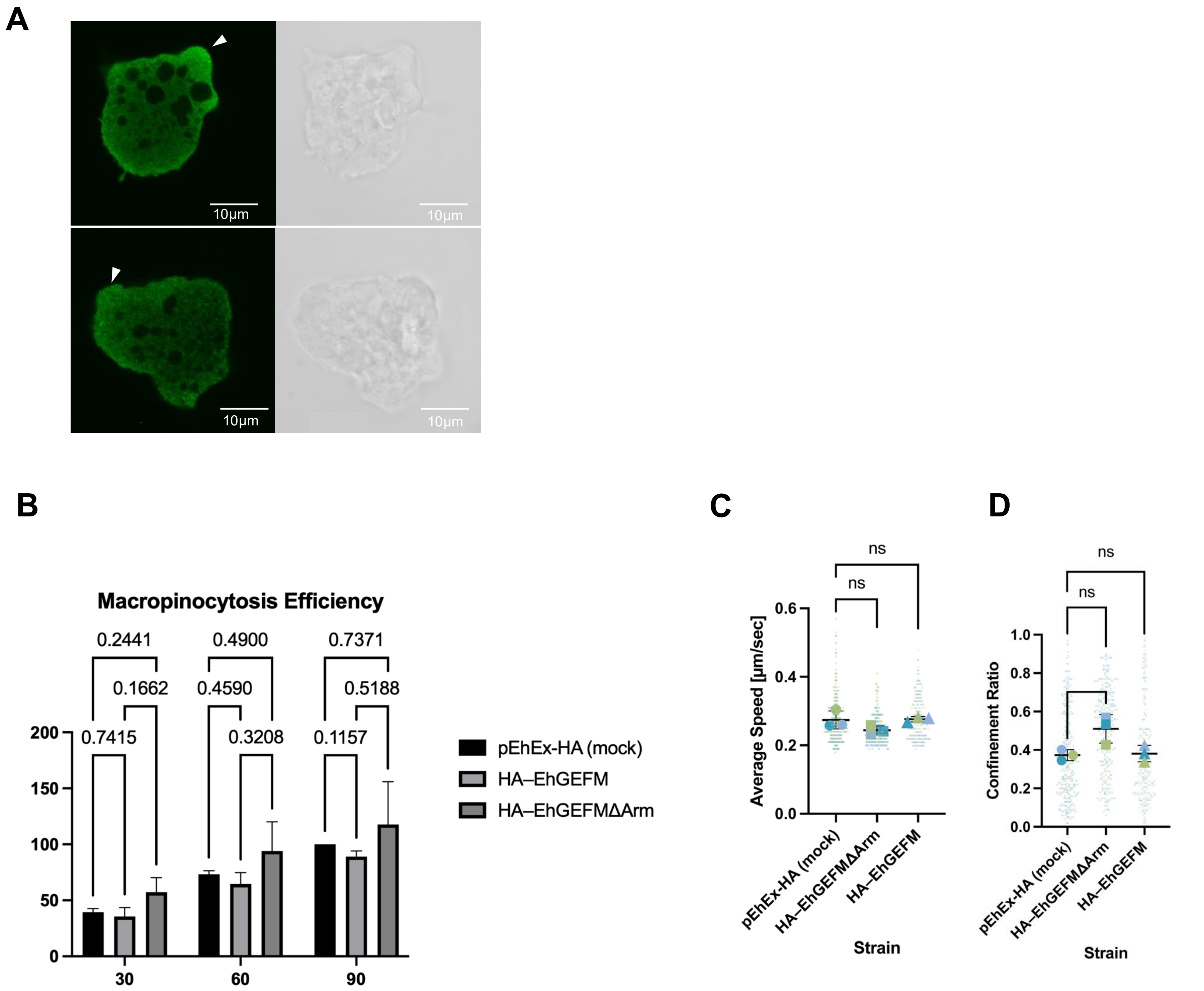

Fig. S4. Localization of HA–EhGEFMΔArm and its effect on macropinocytosis and migration
(A) Immunofluorescence images of representative *E. histolytica* trophozoites from the HA–EhGEFMΔArm-overexpressing strain. The left panels show HA–EhGEFMΔArm visualized using an anti-HA antibody, while the right panels display the corresponding DIC images. White arrowheads indicate HA–EhGEFMΔArm enrichment at pseudopods. **(B)** Macropinocytosis of HA–EhGEFM-overexpressing, HA–EhGEFMΔArm-overexpressing, and mock-transfected (pEhEx-HA) strains was evaluated by FACS. Trophozoites were incubated in RITC–dextran-containing BIS medium, and the fluorescence intensity of RITC–dextran incorporated by each strain was measured as described in Materials and Methods. RITC–dextran incorporation of each strain was estimated by calculating the geometric mean of the fluorescence intensity after subtraction of the background signal from unlabeled parasites in the PE-A channel of each strain and is shown relative to the value of the mock strain at 90 min. Error bars indicate the SD of three biological replicates, and estimated p-values from two-way ANOVA are shown. **(C)** and **(D)** show the results of the motility assay of HA–EhGEFM- and HA–EhGEFMΔArm-overexpressing strains, representing average speed **(C)** and confinement ratio **(D)**. Each small, semi-transparent dot corresponds to the motility value of a single trophozoite, color-coded by biological replicate. Larger dots indicate the mean value of each replicate, using the same color scheme. Bars represent the mean of three biological replicates, and error bars indicate the s.d. Statistical significance was examined using an unpaired two-tailed t-test (ns: not significant).

Journal of Cell Science • Supplementary information

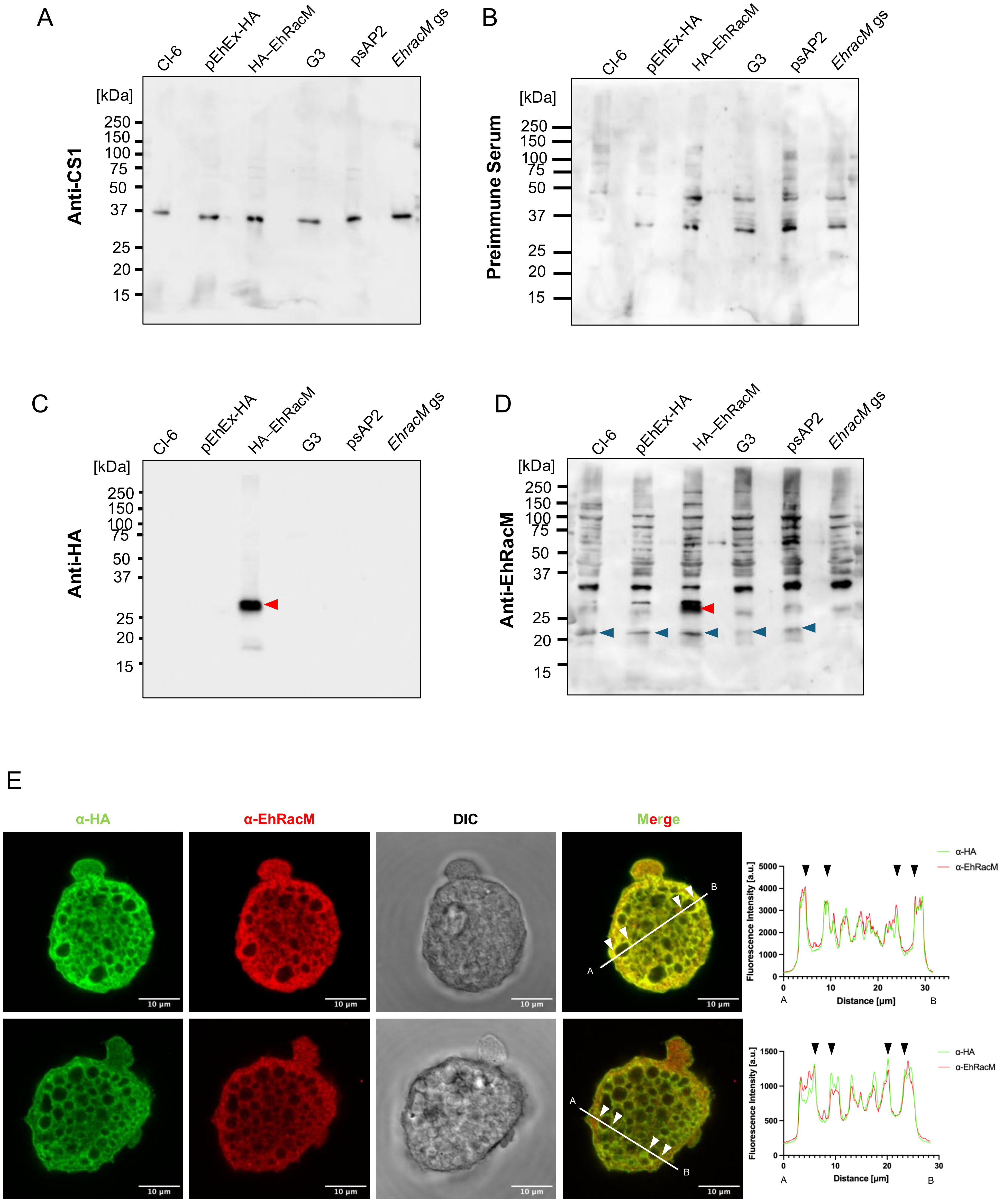
A
Cl-6
pEhEx-HA
HA–EhRacM
G3
psAP2
EhracM gs
[kDa]
250
150
100
75
50
37
25
20
15
Anti-CS1
B
Preimmune Serum
C
Anti-HA
D
Anti-EhRacM
E
α-HA
α-EhRacM
DIC
Merge
10 µm
A
B
Fluorescence Intensity [a.u.]
Distance [µm]

F

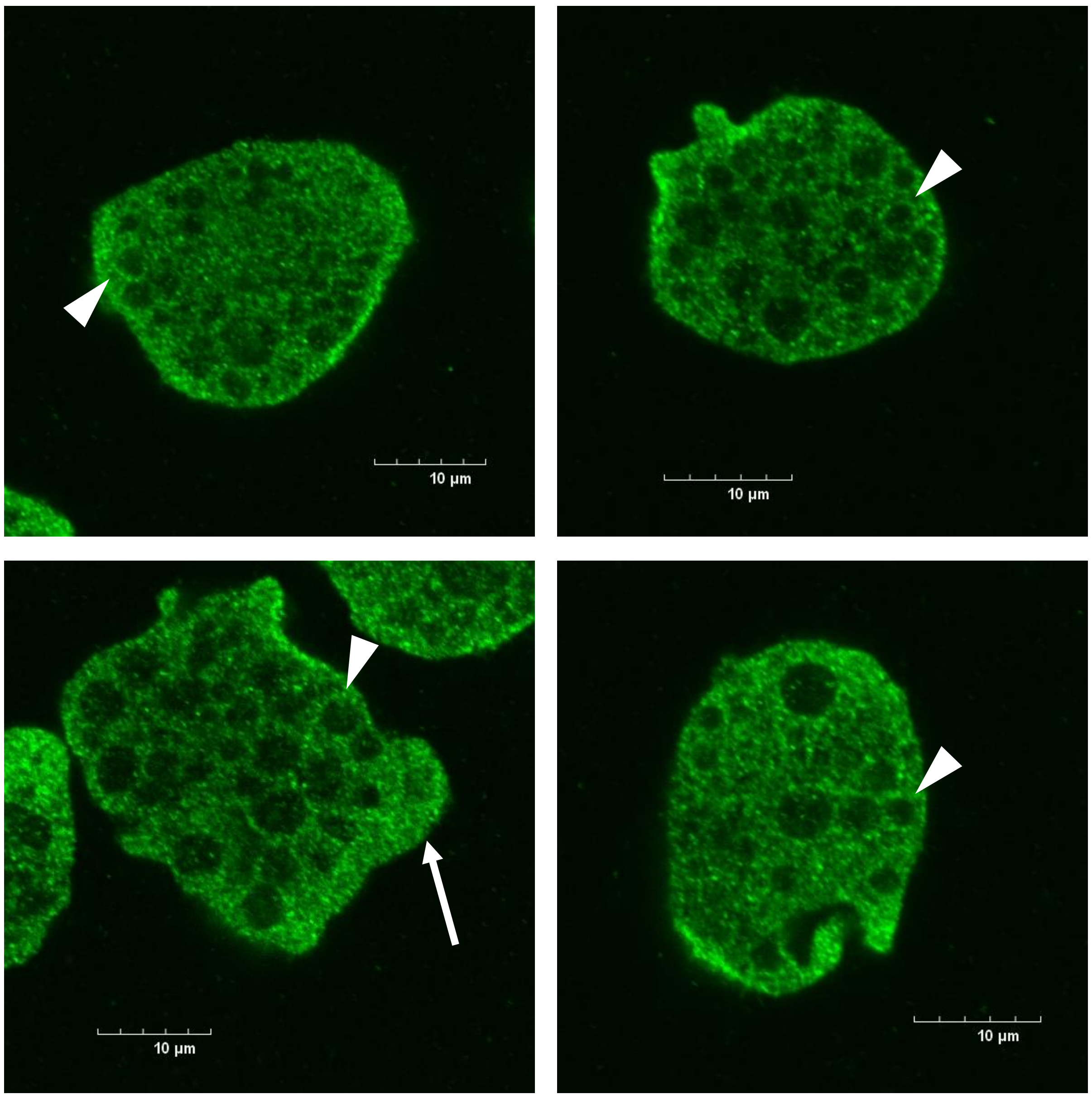

Fig. S5. Validation of the anti-EhRacM antibody. (A) to **(D)** show immunoblot detection of EhRacM in *E. histolytica* parental and the transformant strains using an anti-EhRacM antibody. Approximately 30 µg of total lysates from HM-1:IMSS cl-6 (parental strain), pEhEx-HA (HA mock), HA-fused EhRacM-overexpressing transformant, G3, psAP2 (gene-silencing mock), and EhRacM gene-silenced strains were subjected to SDS-PAGE and immunoblot analysis. The blots were probed with anti-CS1 polyclonal antibody (loading control), anti-HA monoclonal antibody, preimmune serum, and anti-EhRacM polyclonal antibody. Red arrows indicate HA-EhRacM, and blue arrows indicate endogenous EhRacM. **(E)** Immunofluorescence images of representative *E. histolytica* trophozoites from the HA–EhRacM overexpressing strain stained with anti-HA and anti-EhRacM antibodies. The first column shows HA–EhRacM visualized using an anti-HA antibody (green), the second column shows EhRacM stained with an anti-EhRacM antibody (red), the third column shows corresponding DIC images, the fourth column shows merged images of the anti-HA and anti-EhRacM staining, and the fifth column shows fluorescence intensity profiles along the lines drawn in the merged images (from A to B). Black arrowheads correspond to white arrowheads in the intensity plots, indicating vesicle surfaces. Scale bar: 10 µm. **(F)** Localization of endogenous EhRacM detected by anti-EhRacM antibody. Each of the four panels shows the staining of the HM-1:IMSS cl-6 strain with anti-EhRacM antibody (green). Scale bar: 10 µm. Arrowheads point to a possible nascent macropinosome, whereas arrows indicate pseudopods.

Journal of Cell Science • Supplementary information

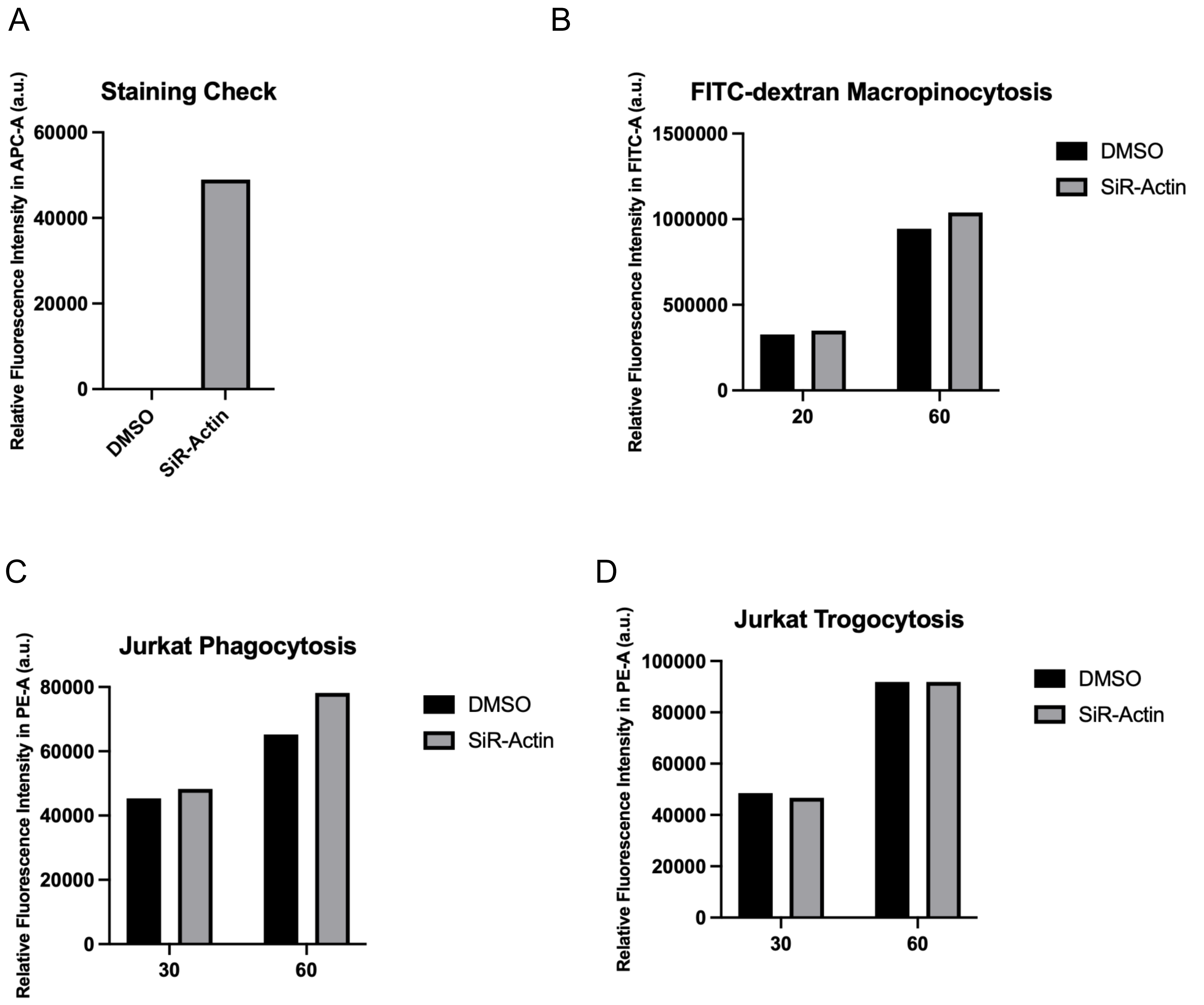

Fig. S6. SiR-Actin staining does not significantly alter endocytic processes in *Entamoeba histolytica*. (A) SiR-Actin staining was confirmed by comparing fluorescence intensity between cells incubated in SiR-Actin-containing medium and those in DMSO-containing medium (control). **(B)** Macropinocytosis was quantified in the presence of SiR-Actin or DMSO (control). Trophozoites of HM-1:IMSS cl-6 were incubated in BIS medium containing FITC–dextran for 20–60 min. **(C)** Phagocytosis of Jurkat cells was quantified in the presence of SiR-Actin or DMSO (control). Trophozoites of HM-1:IMSS cl-6 were incubated in BIS medium with CellTracker™ Orange-stained, pre-heat-killed Jurkat cells for 30–60 min. **(D)** Trogocytosis of Jurkat cells was quantified in the presence of SiR-Actin or DMSO (control). Trophozoites of HM-1:IMSS cl-6 were incubated in BIS medium with CellTracker™ Orange-stained live Jurkat cells for 30–60 min. Fluorescence was measured using a BD Accuri™ C6 Plus flow cytometer, and data were analyzed using FlowJo software. Geometric mean fluorescence intensity in the APC-A **(A)**, FITC-A **(B)**, and PE-A **(C, D)** channels is shown.

A

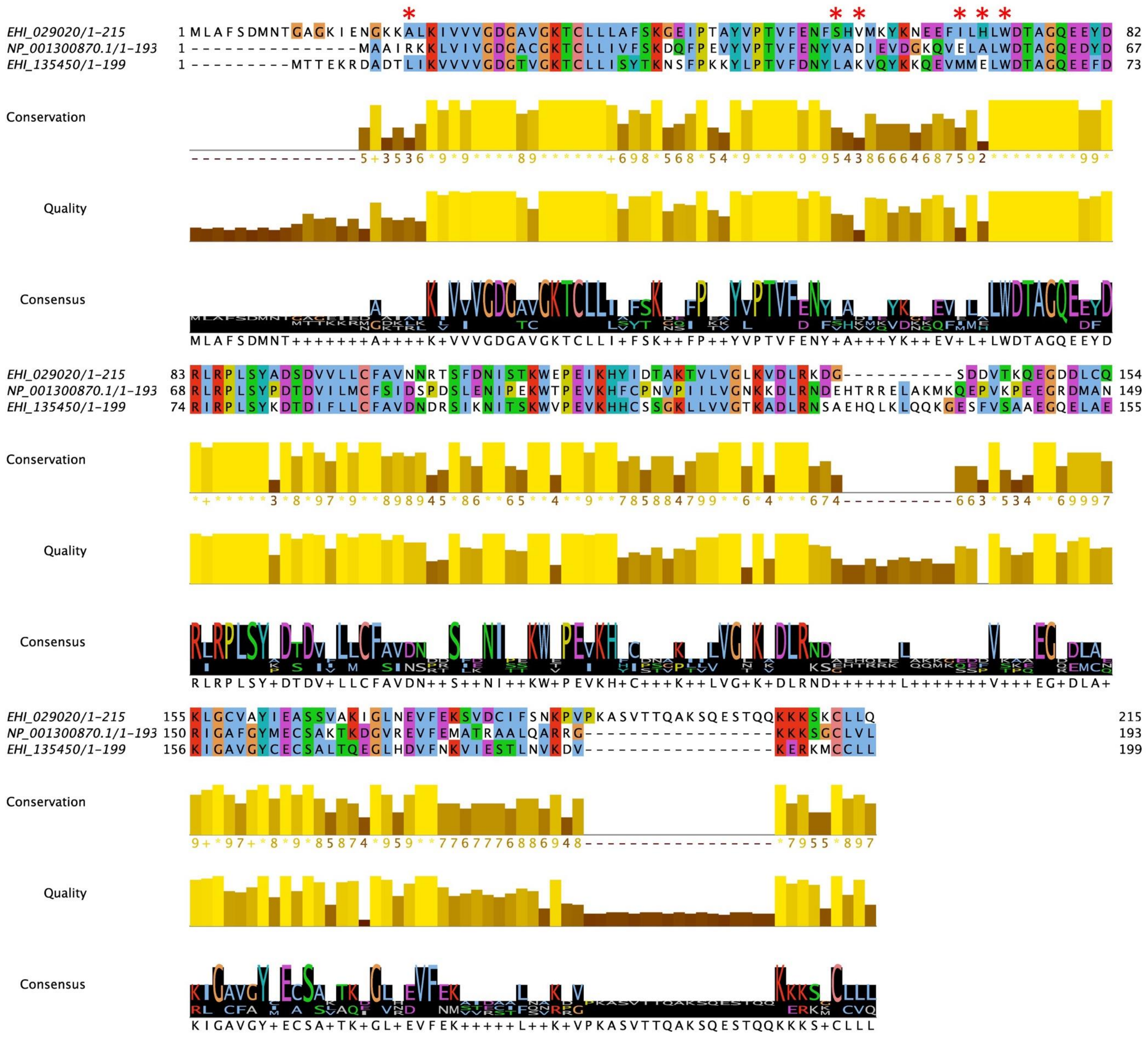

Journal of Cell Science • Supplementary information

B

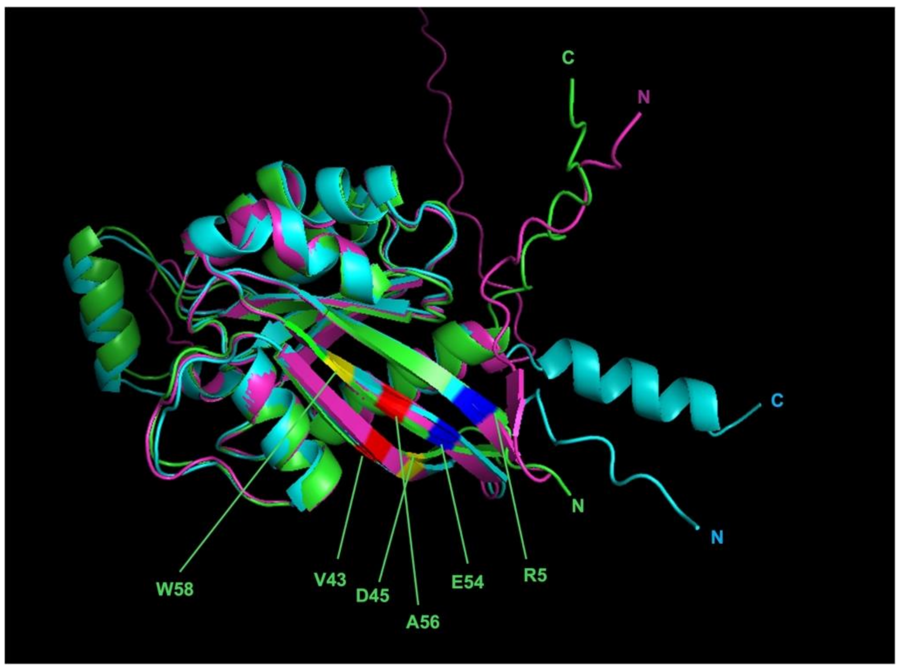

C

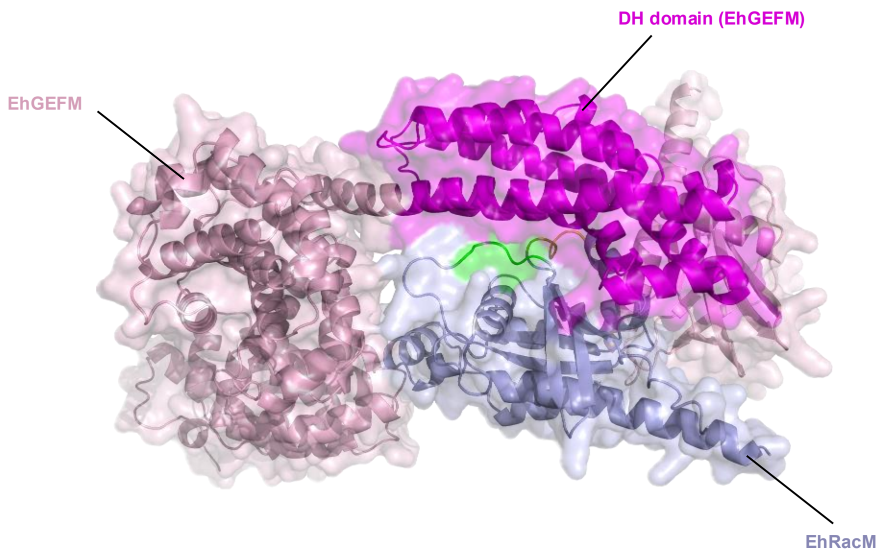

D

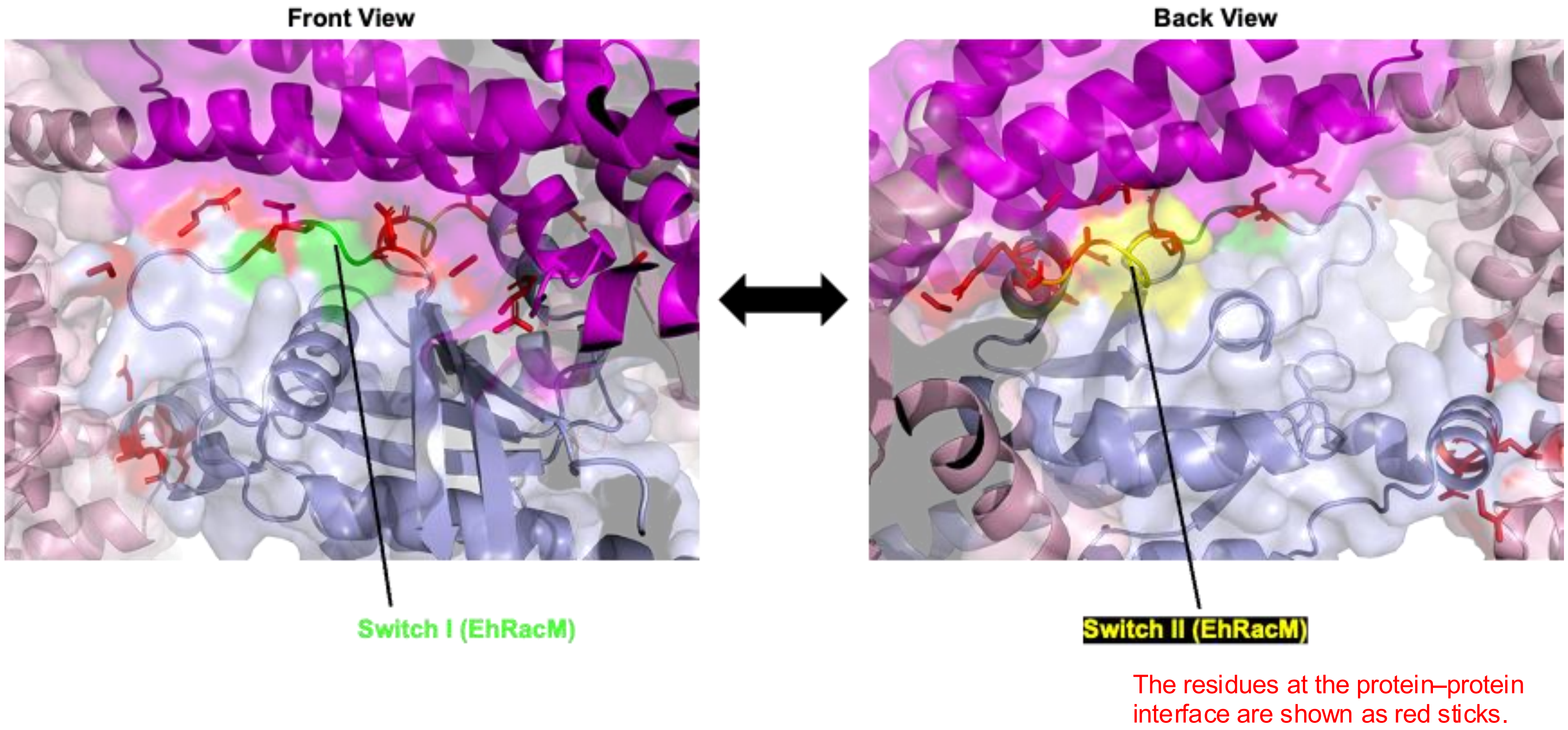

Fig. S7. Rho residues predicted to interact with the DH domain. (A) Multiple Sequence Alignment (MSA) of HsRhoA (NP_001300870.1), EhRho1B (EHI_029020), and EhRacM (EHI_135450) by Clustal Omega. Residues predicted to interact with the DH domain are highlighted with red asterisks, based on RhoA–RhoGEF interaction. Amino acid conservation ratio, alignment quality, and consensus residues are shown. **(B)** AlphaFold2-predicted 3D structures of Rho GTPases were aligned using PyMOL. HsRhoA (UniProt: P61586) is shown in green, *Eh*Rho1B (C4M4W4) in magenta, and *E. histolytica* RacM (C4M9S3) in cyan. “N” and “C” indicate the N- and C-termini, respectively. The C-terminus of EhRacM is not displayed due to space constraints. Key residues in RhoA known to interact with the DH domain—Arg5 (R5), Val43 (V43), Asp45 (D45), Glu54 (E54), Ala56 (A56), and Trp58 (W58)—are highlighted in blue, red, yellow, blue, red, and yellow, respectively. The corresponding residues in EhRho1B and EhRacM are highlighted using the same color scheme. **(C)-(D)** AlphaFold2 multimer model of the EhGEFM–EhRacM complex. **(C)** Overall view showing the DH domain (magenta) of EhGEFM (lightpink) engaging the Switch I (green) and Switch II (yellow) regions of EhRacM (lightblue). The model predicts secondary contacts between the Arm domain of the EhGEFM and the Rho insert region of EhRacM (residues 130–135). **(D)** Close-up view of the interface between the EhGEFM and EhRacM, with residues at the protein–protein interface shown as red sticks. These residues represent atoms within 4 Å between EhRacM and EhGEFM in the predicted model.

Journal of Cell Science • Supplementary information

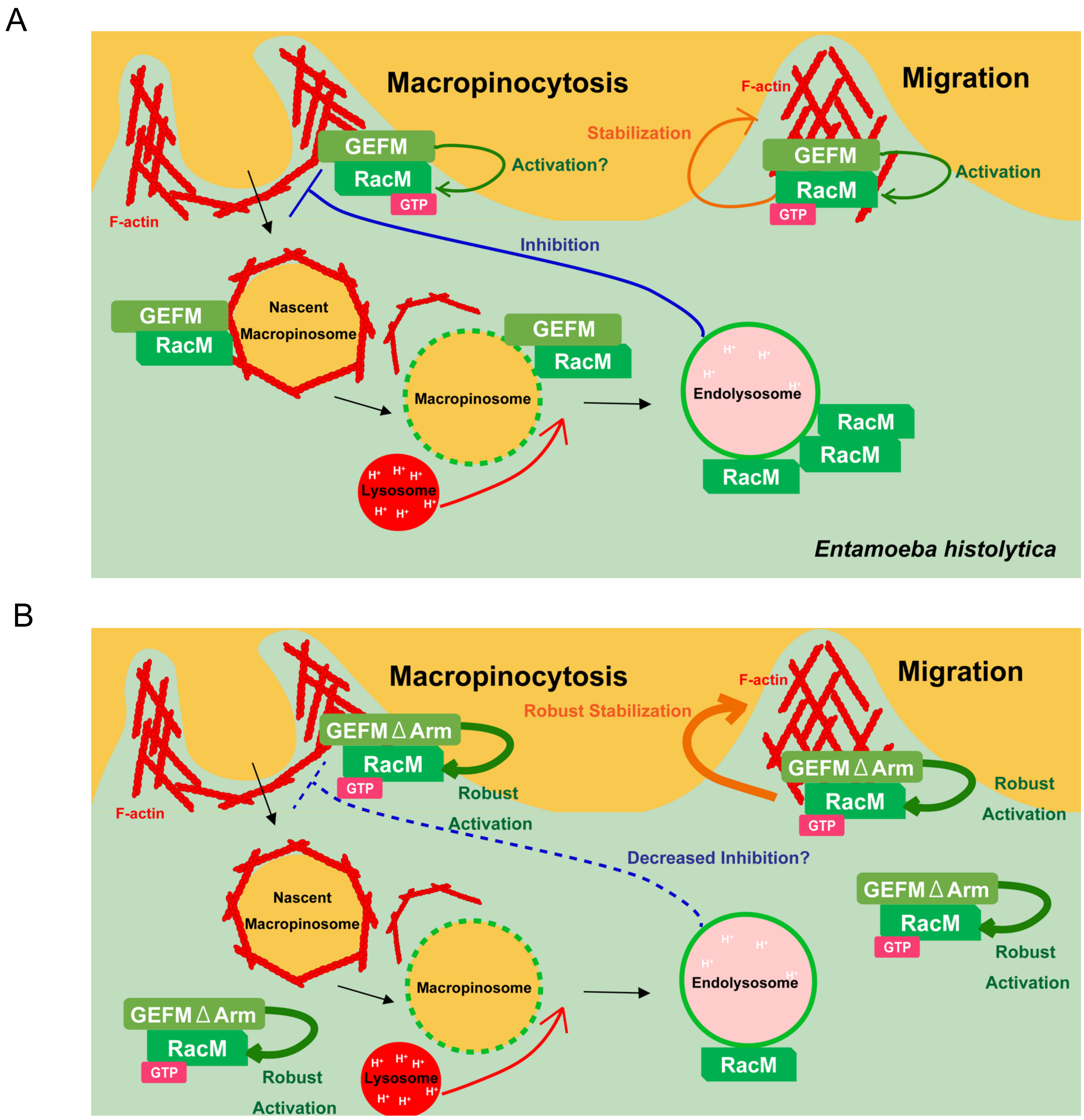

Fig. S8. Hypothetical model of EhGEFM and EhRacM in macropinocytosis and migration. Schematic comparison of full-length EhGEFM **(A)** and the Arm repeat-deleted mutant (EhGEFMΔArm) **(B)**. EhGEFM (light green) is proposed to activate EhRacM (green) at the cell periphery, particularly in pseudopods, thereby promoting linear and directional migration. Consistent with this model, silencing of *EhgefM* or *EhracM* impaired directional persistence, likely due to reduced pseudopod stability or the formation of randomly oriented protrusions.

HA–EhGEFM was also recruited to macropinocytic cups, where it may activate EhRacM during the early stage of cup formation. However, since *EhgefM* silencing did not significantly affect macropinocytosis, this early activation appears to be dispensable for the overall progression of macropinocytosis. In contrast, EhRacM predominantly accumulates on macropinosome membranes after disassembly of the F-actin coat (Shimoyama et al., 2024), suggesting a later role that may be regulated by alternative upstream signals.

Given that overexpression of HA–EhGEFMΔArm caused a slight increase in directional migration, and that EhGEFMΔArm exhibits higher activity toward EhRacM than the full-length protein, the EhGEFM–EhRacM axis might regulate F-actin stability in pseudopods. The slight increase in macropinocytosis upon HA–EhGEFMΔArm overexpression might result from ectopic activation of EhRacM in the cytosol and disruption of the negative signal from mature macropinosomes/endolysosomes to macropinocytosis initiation. These possibilities require further investigation.

Journal of Cell Science • Supplementary information

A

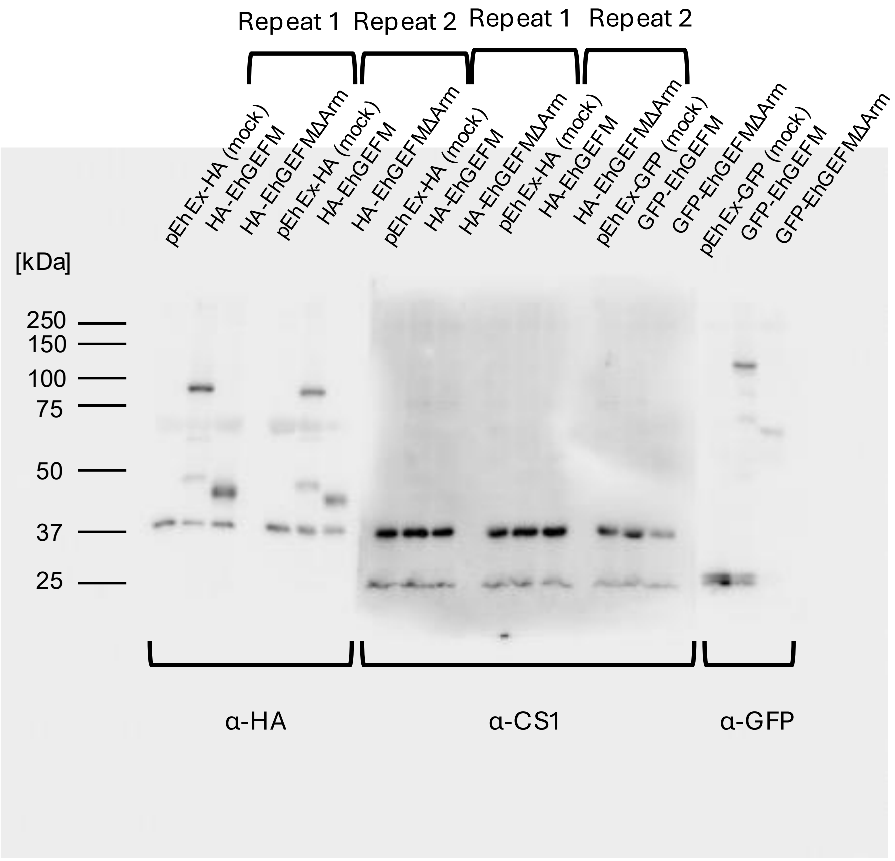

B

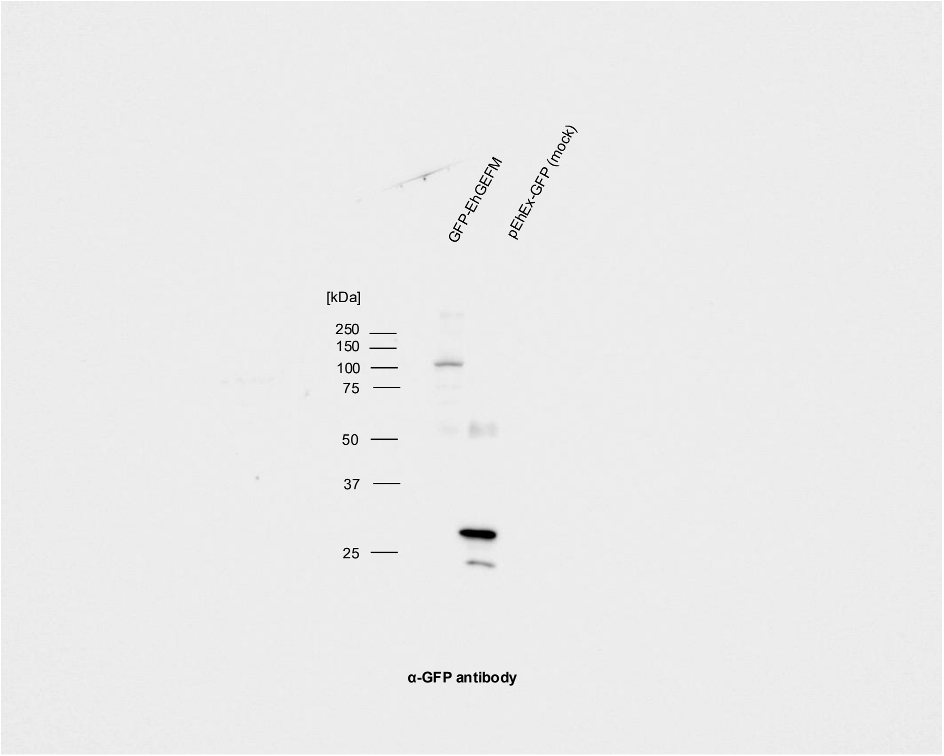

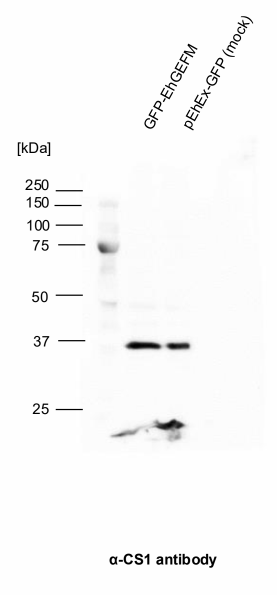

Journal of Cell Science • Supplementary information

C

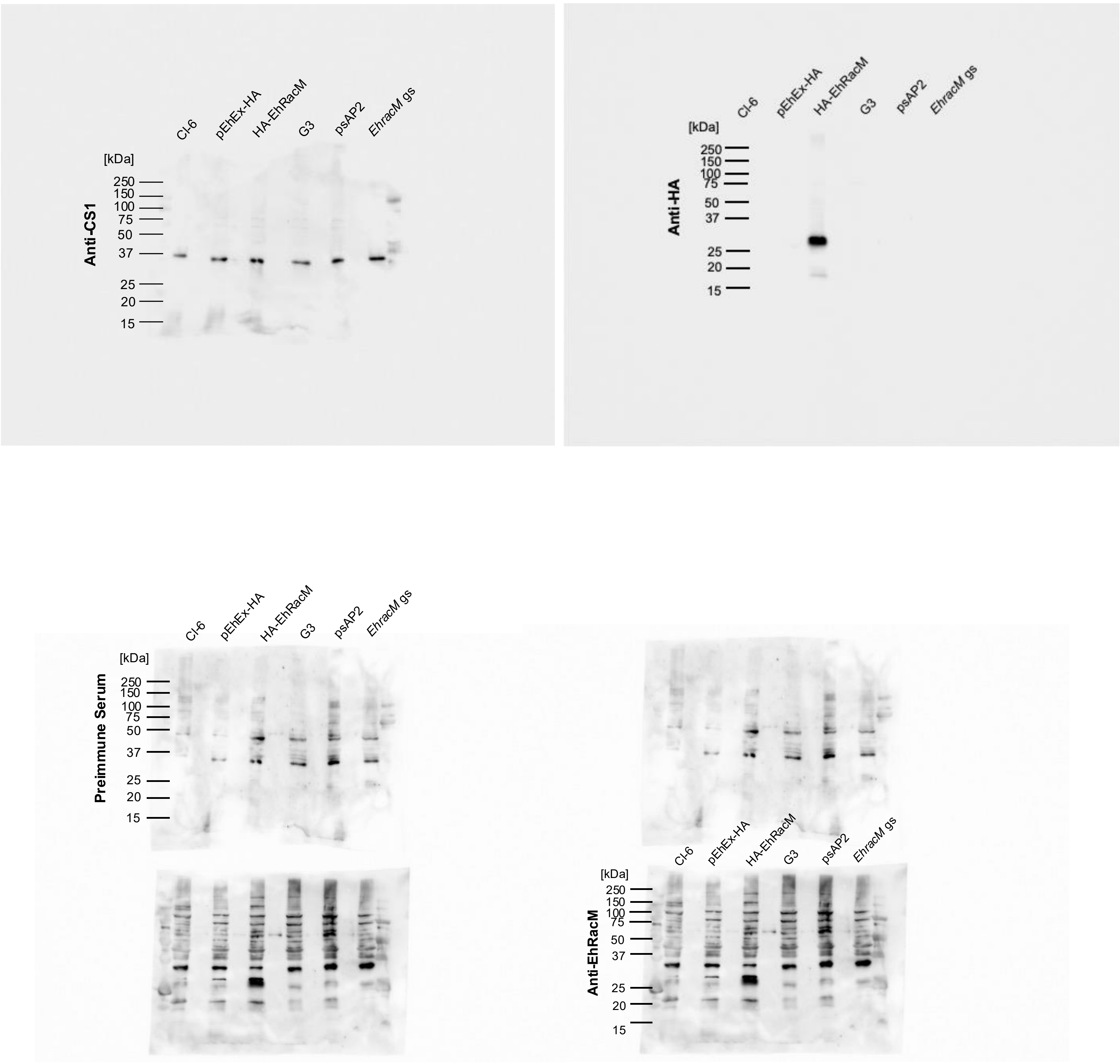

Fig. S9. Blot transparency. (A) Fig. 4A. **(B)** Fig. 4C. **(C)** Fig. S5A. The bottom two show the same source image, with each panel highlighting a different region of the blot for clarity.

Journal of Cell Science • Supplementary information

Table S1. Amino acid sequences of EhGEFM and EhRho/Rac proteins used in this study

This table lists the amino acid sequences and annotation names of EhGEFM and EhRho/Rac proteins used in this study. The region of the EhGEFM sequence covered by the EhGEFMΔArm construct is highlighted in red.

Available for download at
https://journals.biologists.com/jcs/article-lookup/doi/10.1242/jcs.264490#supplementary-data

Table S2. List of primers used in this study

Restriction sites are marked by bold letters. S stands for sense strand, whereas AS stands for antisense strand.

Available for download at
https://journals.biologists.com/jcs/article-lookup/doi/10.1242/jcs.264490#supplementary-data

Table S3. List of hits identified by HA–EhGEFM co-IP

This table lists the hits identified in HA–EhGEFM co-IP, where the mean quantitative value (QV) from two replicates of the co-IP experiment is greater than that of the mock strain. The columns "HA–EhGEFM_1", "mock_1", "HA–EhGEFM_2", and "mock_2" represent the QV from the first and second co-IP of the HA–EhGEFM-overexpressing strain co-IP, and the corresponding QV for the mock strain, respectively. The "Average EhGEFM QV" column indicates the average QV from the two co-IPs of the HA–EhGEFM-overexpressing strain, while the "Average mock QV" column shows the mean QV from the mock strain. The "Average HA–EhGEFM QV/mock QV" column represents the ratio of the average HA–EhGEFM QV to the average mock QV. Note that hits from the HA-EhGEFM-overexpressing strain with QVs less than 1/100 of the bait protein (HA–EhGEFM) were excluded from this table. The hits are ordered by the HA–EhGEFM QV/mock QV ratio. Proteins related to Rho signaling are highlighted in red, those related to the actin cytoskeleton in coral pink, those related to the proteasome system in cream yellow, those associated with G proteins in yellow, and ribosome-related proteins in light green. The "Occurrences" column indicates how many times each hit was detected in previous HA–EhRacM co-IP experiments (n=3), where hits were defined by their QV being higher in the HA–EhRacM-overexpressing strain compared to the mock strain (Shimoyama et al., 2024).

Available for download at
https://journals.biologists.com/jcs/article-lookup/doi/10.1242/jcs.264490#supplementary-data

Journal of Cell Science • Supplementary information

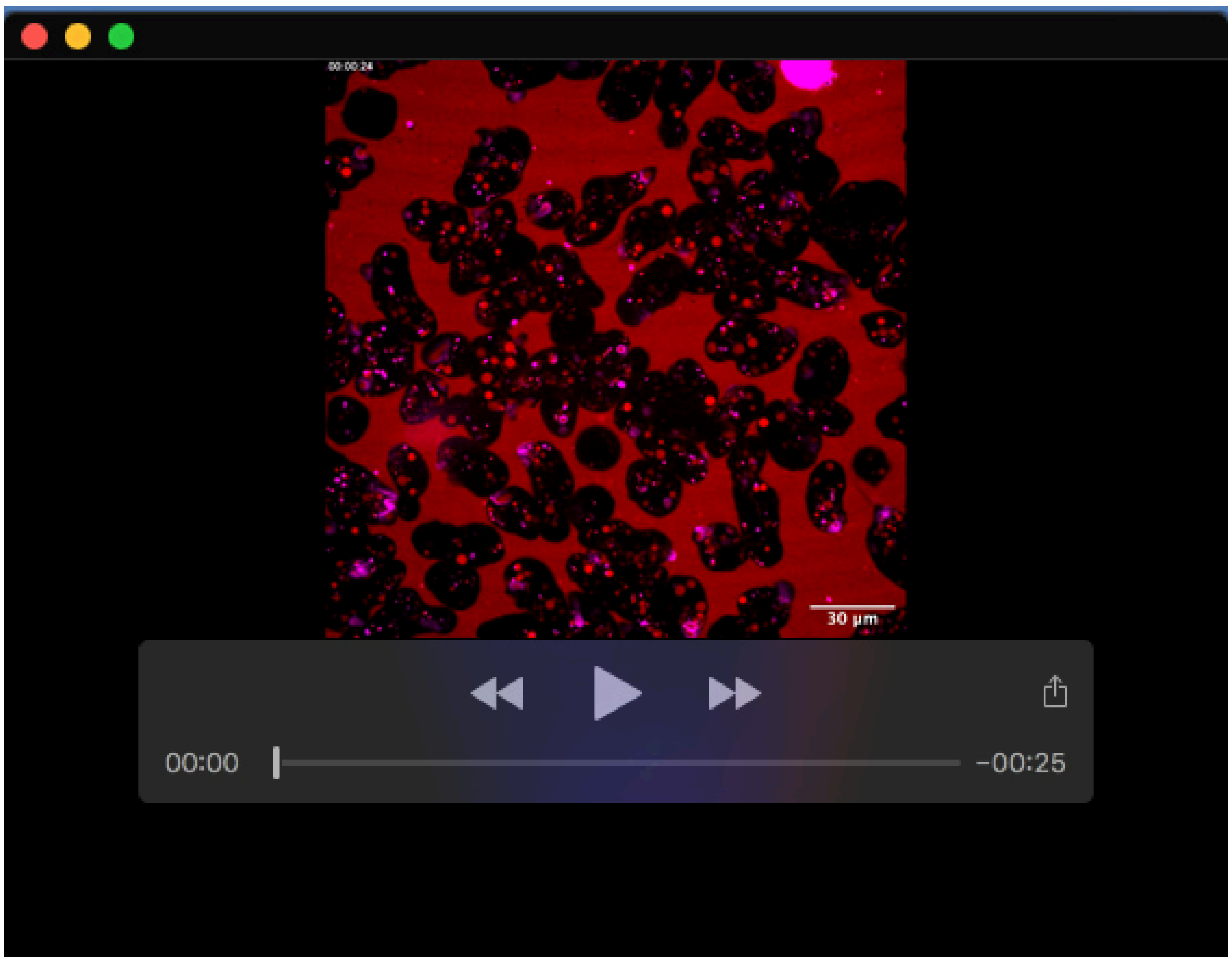

Movie 1. Live imaging of RITC–dextran uptake by *E. histolyitica* trophozoites. Live imaging of *E. histolytica* trophozoites that were incubated with RITC–dextran-containing medium (red). Magenta indicates SiR-Actin staining, which is an F-actin marker. Scale bar: 30 µm.

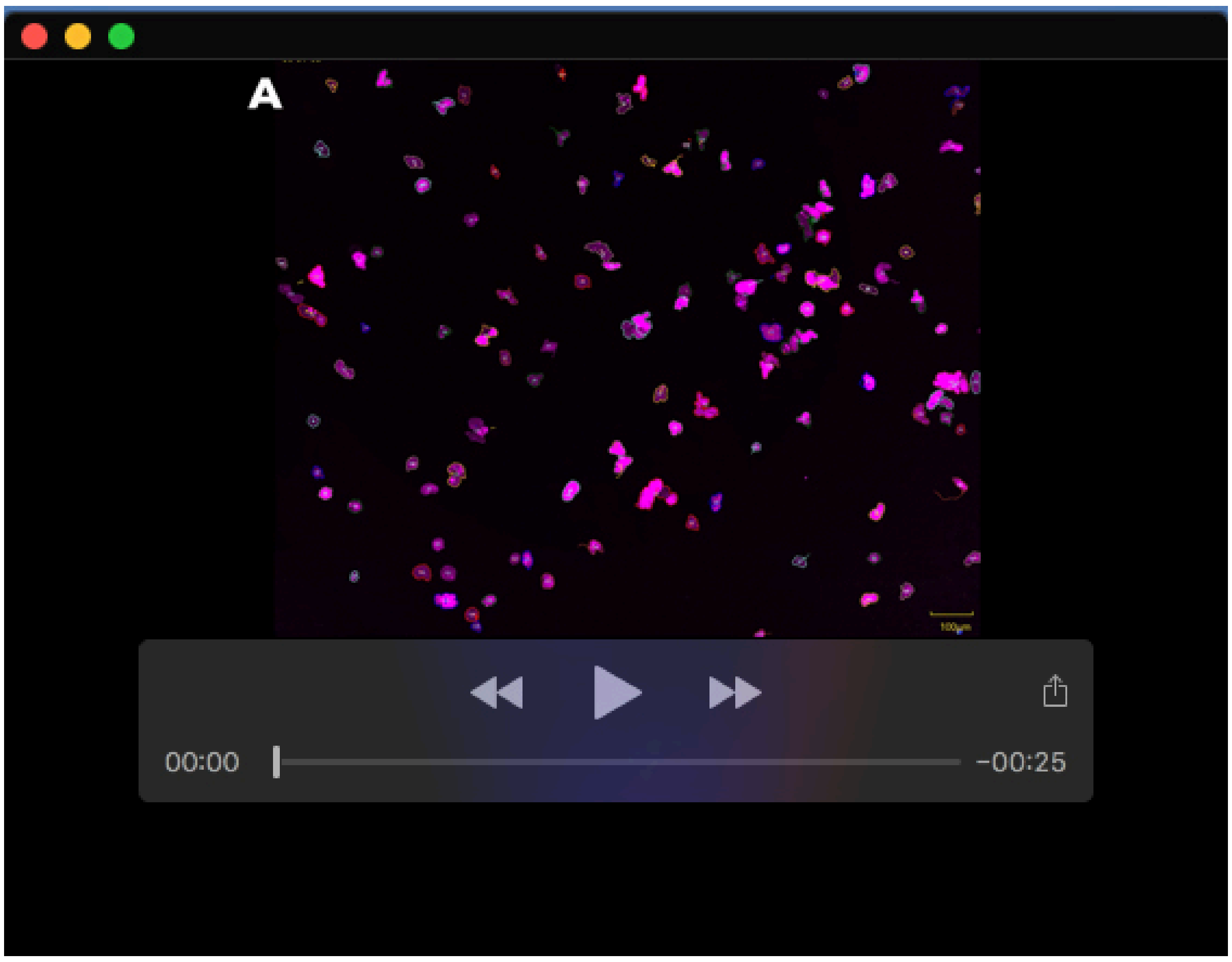

Movie 2. Motility assay using the CQ1 confocal quantitative image cytometer. (A) *EhgefM* gs strain. **(B)** psAP2 (mock) strain. Images were acquired 100 times at 1 s intervals. Scale Bar: 100 µm.

Journal of Cell Science • Supplementary information

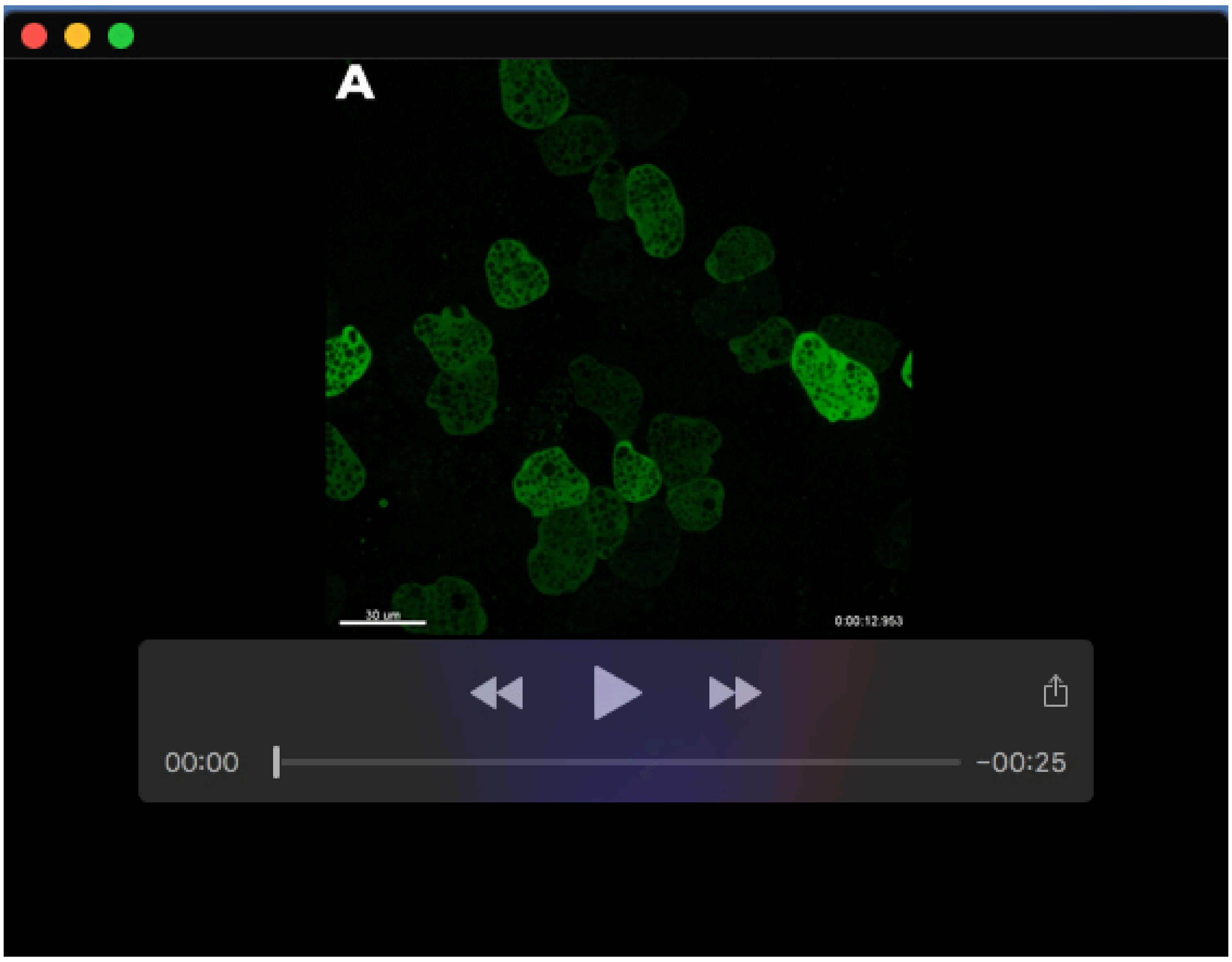

Movie 3. Live imaging of GFP–EhGEFM and GFP (mock). Live imaging of GFP–EhGEFM-overexpressing trophozoites **(A)** and pEhEx-GFP (mock) **(B)** in BIS medium.

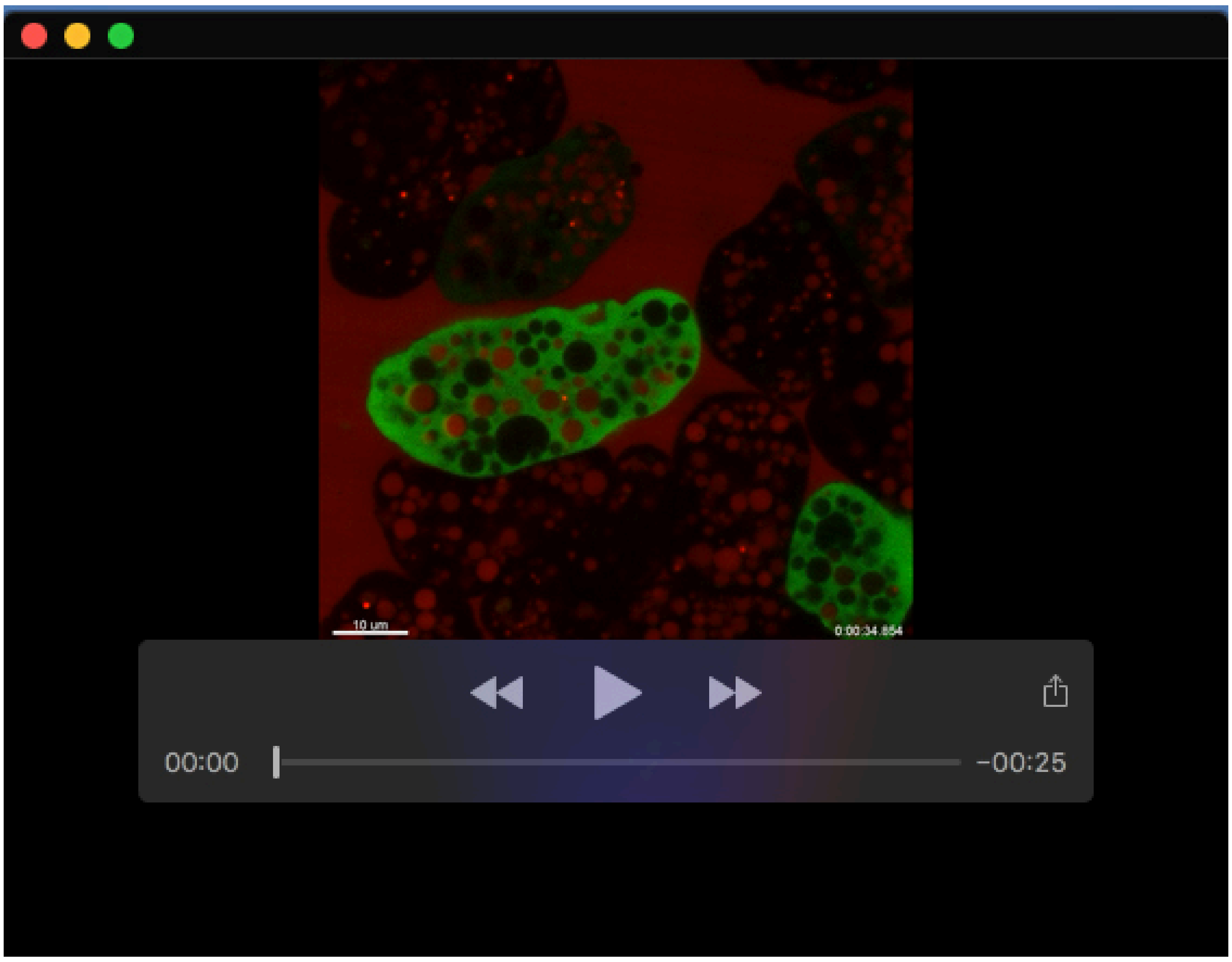

Movie. 4. Localization of GFP–EhGEFM during the initiation of macropinocytosis. Live imaging of GFP–EhGEFM (green) expressing trophozoites that were incubated with RITC–dextran-containing medium (red). Bars, 10 µm.

Journal of Cell Science • Supplementary information

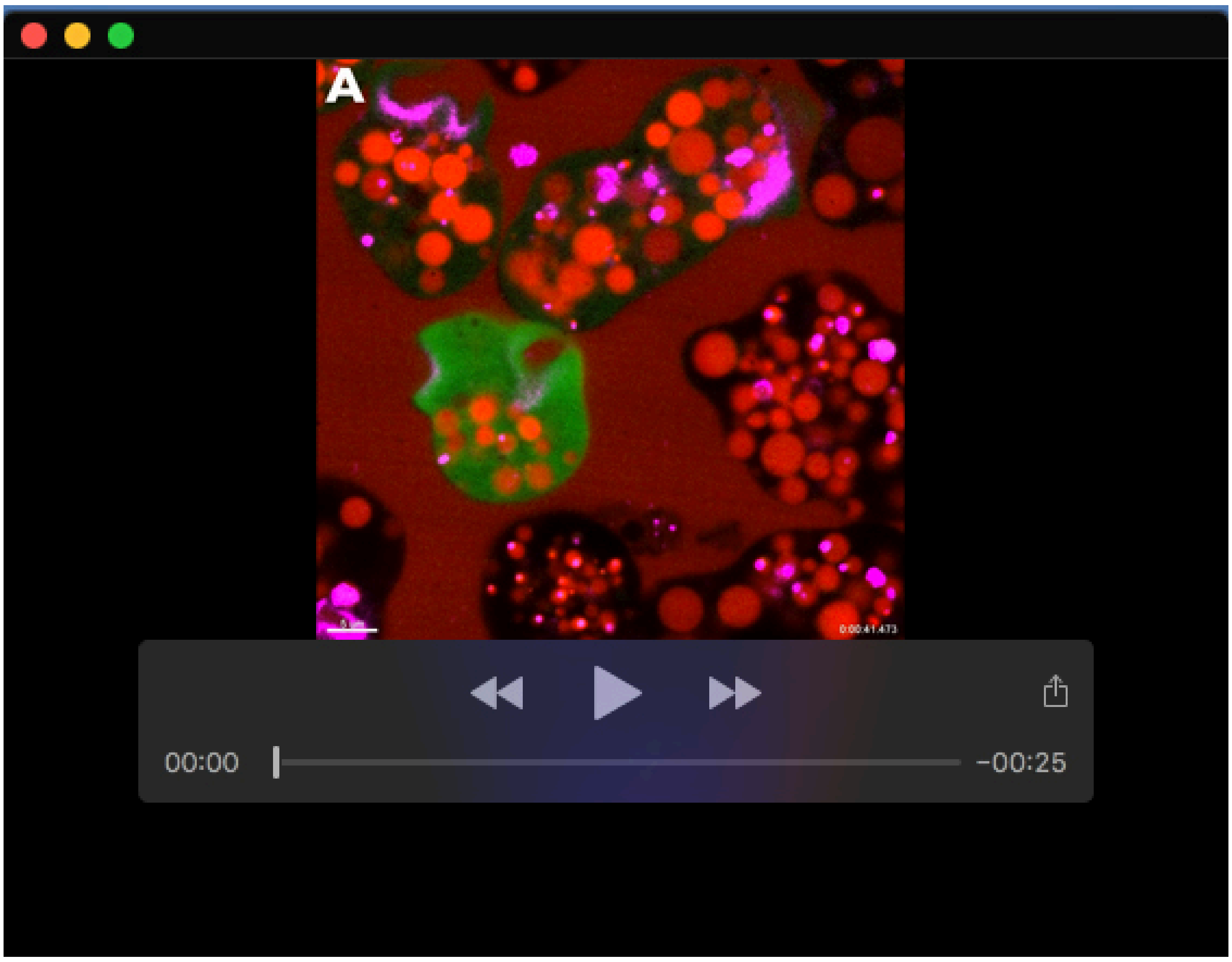

Movie. 5. Localization of GFP–EhGEFM and F-actin during macropinocytosis. Live imaging of GFP–EhGEFM (green) expressing trophozoites that were incubated with RITC–dextran-containing medium (red). Magenta indicates SiR-Actin staining, which is an F-actin marker. Scale bar: 5 µm.

Journal of Cell Science • Supplementary information
