# Supplementary material for: A RhoGEF activates RacM to selectively promote motility without affecting macropinocytosis in Entamoeba histolytica
Source: J Cell Sci. 2026 Jun 16;139(11):jcs264490. doi: 10.1242/jcs.264490 (PMC13327543; doi:10.1242/jcs.264490)
Supplement: Supplementary file 1 [file joces-139-264490_review_history.pdf]

Journal of Cell Science

A RhoGEF activates RacM to selectively promote motility without affecting macropinocytosis in *Entamoeba histolytica*

Misato Shimoyama, Kumiko Nakada-Tsukui and Tomoyoshi Nozaki

DOI: 10.1242/jcs.264490

Editor: Guillaume Jaquemet

Review timeline

Original submission:	5 October 2025
Editorial decision:	28 October 2025
First revision received:	15 April 2026
Accepted:	28 April 2026

Original submission

First decision letter

MS ID#: jcs.264490

MS TITLE: A RhoGEF activates EhRacM to selectively promote motility without affecting macropinocytosis in Entamoeba histolytica

AUTHORS: Misato Shimoyama; Kumiko Nakada-Tsukui; Tomoyoshi Nozaki
ARTICLE TYPE: Research Article

Dear Dr Nozaki,

We have now reached a decision on the above manuscript.

As you will see, the reviewers raise a number of substantial criticisms that prevent me from accepting the paper at this stage. They suggest, however, that a revised version might prove acceptable, if you can address their concerns. If you think that you can deal satisfactorily with the criticisms on revision, I would be pleased to see a revised manuscript. We would then return it to the reviewers.

Reviewer 1

This manuscript by Shimoyama et al. investigates the role of a previously uncharacterized Entamoeba histolytica RhoGEF, named EhGEFM (EHI_158230), in regulating the small GTPase EhRacM (EhRho13), identified through an interactome analysis using EhGEFM as bait. The authors demonstrate that EhGEFM interacts with EhRacM and exhibits guanine nucleotide exchange activity. Silencing of EhgefM did not affect macropinocytosis but impaired directional migration. Using immunofluorescence assays and live-cell imaging, the authors show that EhGEFM colocalizes with EhRacM at the cell periphery and transiently associates with developing macropinocytic cups. Overall, this study provides important insights into the role of EhGEFM in E. histolytica by identifying its regulation of EhRacM and offering the first direct evidence for distinct regulatory pathways controlling Rho small GTPase functions in the parasite, highlighting EhGEFM as a key regulator of EhRacM-dependent directional migration.
The study is technically sound and employs a well-balanced combination of interactome analysis, structural modeling, biochemical assays, and live-cell imaging to explore the mechanism of

© 2026. Published by The Company of Biologists under the terms of the Creative Commons Attribution License (https://creativecommons.org/licenses/by/4.0/).

EhGEFM-EhRacM interaction in depth. The results are clearly presented, and the high-quality imaging data strongly support the study's conclusions.
Silencing of EhGEFM is monitored by RT-PCR and qPCR, which is acceptable for this study; however, confirmation of reduced EhGEFM protein levels would have further strengthened the findings. The authors propose that the Arm domain acts as an autoinhibitory region, but this conclusion is based solely on in vitro assays using truncated proteins. Generating and analyzing Arm-deleted mutants expressed in trophozoites, examining both their localization and phenotype, would provide stronger experimental support for this proposed mechanism.
Supplementary Figure 5 legend: Please include the full reference for EHI_153510 (EhGEF2, identified by Sunando).

Reviewer 2

SUMMARY OF THE ADVANCE MADE IN THIS PAPER AND ITS POTENTIAL SIGNIFICANCE TO THE FIELD

This manuscript investigates the role of RhoGEFM, one of 62 Rho guanine nucleotide exchange factors (RhoGEFs) identified in Entamoeba histolytica. RhoGEFs activate Rho family small GTPases and regulate actin-related cellular processes such as migration and macropinocytosis. The authors previously identified RacM, a small GTPase involved in these phenomena (Narooka et al., 2022) and now explore its interaction with RhoGEFM.

The current study provides evidence that the GEFM-RacM axis contributes specifically to directional motility, but not to macropinocytosis, in E. histolytica. Overall, the topic is interesting and relevant to readers of Journal of Cell Science, as it advances understanding of amoebic cytoskeletal regulation and cell migration.

Overall Assessment
This manuscript extends the authors' prior work on RacM and provides valuable insights into amoebic motility mechanisms. The experimental approach is technically sound and combines omics, biochemistry and imaging techniques effectively. However, the manuscript's length, missing methodological details, and lack of certain critical controls weaken its impact. Addressing these points will significantly improve the clarity, rigor, and readability of the study.

SUGGESTIONS TO AUTHORS
Major Comments
1. Redundancy and manuscript length
The manuscript is considerably long. Since the experimental procedures are essentially identical to those described in the authors' previous publication on RacM, the Materials and Methods section could be significantly shortened by referencing that article.
The Discussion section is also diffuse and would benefit from refocusing on the direct findings of this study. Streamlining these sections would enhance clarity and impact.
2. Role of RhoGEFM in motility vs. macropinocytosis
The central claim—that RhoGEFM regulates cell motility but not macropinocytosis—requires more robust evidence.
o The description of macropinocytosis quantification lacks key details. The dextran molecular weight used for uptake assays is not specified (neither in the previous study).
o It remains unclear how simple endocytosis was excluded from the analysis.
o The inclusion of F-actin staining as a confocal microscopy control would be highly informative, as it reliably distinguishes macropinocytosis from other uptake processes.
3. Validation of live-cell imaging with SiR-actin
The use of SiR-actin in E. histolytica warrants further validation, as previous studies have reported difficulties with this probe in amoebae. Two issues should be addressed explicitly:
a. High background fluorescence (similar to GFP) likely arises from the parasite's intrinsic green autofluorescence.
b. The concentration used (10 μM) is substantially above the recommended limit (1 μM). SiR-actin is based on jasplakinolide, which can inhibit actin dynamics. The authors should demonstrate that this concentration does not alter cytoskeletal behaviour, for instance by testing bacterial or erythrocyte phagocytosis.
4. Microscopy presentation and controls

© 2026. Published by The Company of Biologists under the terms of the Creative Commons Attribution License (https://creativecommons.org/licenses/by/4.0/).

Figures showing colocalization among GEFM (GFP), FITC-dextran, and SiR-actin would be more interpretable if the individual fluorescence channels were presented separately (e.g., in Figure 6). This would help readers evaluate signal overlap and localization accuracy. For colocalization data an index must be calculated (Pearson)

5. Supplementary Figure 6

The image quality in Supplementary Figure 6 requires improvement. The background fluorescence is excessive, making it difficult to discern the colocalization between endogenous RacM and macropinosomes. As this figure supports a key claim, enhancing image clarity is essential.

6. Motility assay analysis (CellPathfinder)

The explanation of the motility parameters requires clarification. Specifically, the rationale for including "total distance" as a relevant metric is unclear, given that it is similar between strains, whereas "confinement" and "net distance" differ. The authors should clarify how each parameter contributes to understanding motility behaviour.

Major comments [Please request additional experiments only if they are essential for supporting the conclusions; authors should be encouraged to highlight any claims that are preliminary or speculative, or to discuss any pitfalls or alternative interpretations in a 'Limitations' section]

Minor comments

First revision

Author response to reviewers' comments

Point-to-point response

We thank the reviewers for their insightful and constructive comments. We have carefully revised the manuscript in accordance with the suggestions and provide point-by-point responses below. We have attached both the cleaned and tracked-changes versions of the manuscripts. The page numbers indicated in this document refer to the cleaned text.

Reviewer 1

This manuscript by Shimoyama et al. investigates the role of a previously uncharacterized Entamoeba histolytica RhoGEF, named EhGEFM (EHI_158230), in regulating the small GTPase EhRacM (EhRho13), identified through an interactome analysis using EhGEFM as bait. The authors demonstrate that EhGEFM interacts with EhRacM and exhibits guanine nucleotide exchange activity. Silencing of EhgefM did not affect macropinocytosis but impaired directional migration. Using immunofluorescence assays and live-cell imaging, the authors show that EhGEFM colocalizes with EhRacM at the cell periphery and transiently associates with developing macropinocytic cups. Overall, this study provides important insights into the role of EhGEFM in E. histolytica by identifying its regulation of EhRacM and offering the first direct evidence for distinct regulatory pathways controlling Rho small GTPase functions in the parasite, highlighting EhGEFM as a key regulator of EhRacM-dependent directional migration.

The study is technically sound and employs a well-balanced combination of interactome analysis, structural modeling, biochemical assays, and live-cell imaging to explore the mechanism of EhGEFM-EhRacM interaction in depth. The results are clearly presented, and the high-quality imaging data strongly support the study's conclusions.

Silencing of EhGEFM is monitored by RT-PCR and qPCR, which is acceptable for this study; however, confirmation of reduced EhGEFM protein levels would have further strengthened the findings.

Response:

We thank the reviewer for this suggestion and agree that direct confirmation of reduced EhGEFM protein levels would strengthen the conclusions. However, as an antibody against EhGEFM is not yet available, direct assessment of protein levels in the silenced strain is currently not feasible.

© 2026. Published by The Company of Biologists under the terms of the Creative Commons Attribution License (https://creativecommons.org/licenses/by/4.0/).

In this study, gene silencing was therefore evaluated at the transcript level by RT-PCR and qPCR, which we consider sufficient to demonstrate reduced expression. Nevertheless, we acknowledge that protein abundance does not always correlate with mRNA levels. This limitation has been noted in the Discussion (lines 427-429): “However, we cannot exclude the possibility that residual EhGEFM protein persists in the silenced strain despite reduced mRNA levels, as protein abundance does not always directly correlate with transcript levels.”

The authors propose that the Arm domain acts as an autoinhibitory region, but this conclusion is based solely on in vitro assays using truncated proteins. Generating and analyzing Arm-deleted mutants expressed in trophozoites, examining both their localization and phenotype, would provide stronger experimental support for this proposed mechanism.

Response:
We thank the reviewer for this suggestion. To address this point, we generated an Arm repeat-deleted mutant of EhGEFM (HA-EhGEFMΔArm) and examined its localization and phenotypes in trophozoites. These data have been incorporated into the revised manuscript (Results, lines 266-294; Discussion, lines 430-448; Figs 4A and S4). Briefly, HA-EhGEFMΔArm expression showed a trend toward increased linear motility and macropinocytosis, although these effects were not statistically significant. The mutant localized predominantly to the cytosol, with enrichment at pseudopods, similar to full-length HA-EhGEFM.

Given that deletion of the Arm repeat is expected to relieve autoinhibition and enhance GEF activity toward EhRacM, these findings are consistent with a role for the EhGEFM-EhRacM axis in regulating cell motility. The modest increase in macropinocytosis upon HA-EhGEFMΔArm overexpression may reflect perturbed signaling, further supported by the reduced growth rate observed in these cells.

Supplementary Figure 5 legend: Please include the full reference for EHI_153510 (EhGEF2, identified by Sunando).

Response:
We thank the reviewer for identifying this error. EHI_153510 was incorrectly labeled as EhGEF2 in the original figure legend; it corresponds to EhFP4. This has been corrected in the revised manuscript.

Reviewer 2

SUMMARY OF THE ADVANCE MADE IN THIS PAPER AND ITS POTENTIAL SIGNIFICANCE TO THE FIELD

This manuscript investigates the role of RhoGEFM, one of 62 Rho guanine nucleotide exchange factors (RhoGEFs) identified in Entamoeba histolytica. RhoGEFs activate Rho family small GTPases and regulate actin-related cellular processes such as migration and macropinocytosis. The authors previously identified RacM, a small GTPase involved in these phenomena (Narooka et al., 2022) and now explore its interaction with RhoGEFM.

The current study provides evidence that the GEFM-RacM axis contributes specifically to directional motility, but not to macropinocytosis, in E. histolytica. Overall, the topic is interesting and relevant to readers of Journal of Cell Science, as it advances understanding of amoebic cytoskeletal regulation and cell migration.

Overall Assessment
This manuscript extends the authors' prior work on RacM and provides valuable insights into amoebic motility mechanisms. The experimental approach is technically sound and combines omics, biochemistry and imaging techniques effectively. However, the manuscript's length, missing methodological details, and lack of certain critical controls weaken its impact. Addressing these points will significantly improve the clarity, rigor, and readability of the study.

SUGGESTIONS TO AUTHORS

© 2026. Published by The Company of Biologists under the terms of the Creative Commons Attribution License (https://creativecommons.org/licenses/by/4.0/).

Major Comments
1. Redundancy and manuscript length
The manuscript is considerably long. Since the experimental procedures are essentially identical to those described in the authors' previous publication on RacM, the Materials and Methods section could be significantly shortened by referencing that article.
The Discussion section is also diffuse and would benefit from refocusing on the direct findings of this study. Streamlining these sections would enhance clarity and impact.

Response:
We thank the reviewer for this suggestion. The Materials and Methods section has been streamlined by removing redundant descriptions and referring to our previous EhRacM study. The Discussion section has also been revised to focus more directly on the findings of the present study, improving clarity and overall readability.

2. Role of RhoGEFM in motility vs. macropinocytosis
The central claim—that RhoGEFM regulates cell motility but not macropinocytosis—requires more robust evidence.
o The description of macropinocytosis quantification lacks key details. The dextran molecular weight used for uptake assays is not specified (neither in the previous study).
o It remains unclear how simple endocytosis was excluded from the analysis.
o The inclusion of F-actin staining as a confocal microscopy control would be highly informative, as it reliably distinguishes macropinocytosis from other uptake processes.

Response:
We thank the reviewer for raising this important point. In *Entamoeba histolytica*, distinguishing macropinocytosis from other endocytic pathways remains technically challenging. Although the genome encodes clathrin, suggesting the presence of receptor-mediated endocytosis, previous work (Welter et al., 2006) showed that both FITC-dextran and transferrin uptake are actin-dependent, underscoring the difficulty of clearly separating these pathways.

Point 1: Dextran molecular weight
Dextran (70kDa) was used for the uptake assays. This has been added to the Materials and Methods section (lines 566-567).

Point 2: Exclusion of receptor-mediated endocytosis
A standardized quantitative method to distinguish receptor-mediated endocytosis from macropinocytosis in *E. histolytica* is not currently available. Fluid-phase dextran uptake is therefore used as a proxy for macropinocytosis in this organism.

Point 3: F-actin staining
In our preliminary experiments using SiR-Actin, bulk fluid-phase uptake events were generally accompanied by actin accumulation (Movie S1). While contribution from other pathways cannot be excluded, the majority of uptake appears consistent with actin-dependent macropinocytosis. This point has been clarified in the Result section (lines 243-246): “In *E. histolytica*, fluid-phase uptake is generally considered to occur predominantly via actin-dependent macropinocytosis (Movie S1), which enables bulk fluid-phase uptake, although clear experimental criteria to distinguish it from other endocytic routes remain limited.”

3. Validation of live-cell imaging with SiR-actin
The use of SiR-actin in E. histolytica warrants further validation, as previous studies have reported difficulties with this probe in amoebae. Two issues should be addressed explicitly:
a. High background fluorescence (similar to GFP) likely arises from the parasite's intrinsic green autofluorescence.
b. The concentration used (10 μM) is substantially above the recommended limit (1 μM). SiR-actin is based on jasplakinolide, which can inhibit actin dynamics. The authors should demonstrate that this concentration does not alter cytoskeletal behaviour, for instance by testing bacterial or erythrocyte phagocytosis.

© 2026. Published by The Company of Biologists under the terms of the Creative Commons Attribution License (https://creativecommons.org/licenses/by/4.0/).

Response:
We thank the reviewer for raising these concerns.

Point a. Autofluorescence and background signal
Background fluorescence is a known challenge in *E. histolytica*. In our previous study (Shimoyama et al., 2024), SiR-Actin staining was validated by co-localization with phalloidin (Fig. S11 in that study). However, consistent visualization of actin structures in live trophozoites required relatively high concentrations (10 μM). In addition, components of the culture medium, including vitamins and 15% adult bovine serum, contribute to background fluorescence. Serum-free conditions reduce background, but impair cell motility and alter normal cellular behavior, making them unsuitable for imaging dynamic processes. To improve signal interpretation, fluorescence intensity line-scan analysis has been added in Fig. 7.

Point b. Potential effects of high SiR-actin concentration
We assessed the impact of SiR-Actin on macropinocytosis, phagocytosis, and trogocytosis in this parasite. As shown in Fig. S6, these processes were comparable between DMSO-treated control cells and SiR-Actin-stained cells, indicating that the staining conditions do not substantially impair actin-dependent cellular processes. This has been clarified in the Results section (lines 347-350): “Note that compatibility of SiR-Actin staining with phalloidin labeling was previously confirmed (Shimoyama et al., 2024). We also verified that SiR-Actin staining under the conditions used here does not significantly affect macropinocytosis, phagocytosis, or trogocytosis (Fig. S6).”

4. Microscopy presentation and controls
Figures showing colocalization among GEFM (GFP), FITC-dextran, and SiR-actin would be more interpretable if the individual fluorescence channels were presented separately (e.g., in Figure 6). This would help readers evaluate signal overlap and localization accuracy. For colocalization data an index must be calculated (Pearson)

Response:

We thank the reviewer for this suggestion. Fig. 7 has been revised to show individual fluorescence channels for GFP-EhGEFM, FITC-dextran, and SiR-Actin. In addition, fluorescence intensity line-scan analysis was performed across representative macropinocytic cups, allowing direct comparison of the signal distributions. Given the localized and dynamic nature of these events, line-scan analysis was considered more appropriate than a global colocalization metric, such as Pearson’s coefficient.

5. Supplementary Figure 6
The image quality in Supplementary Figure 6 requires improvement. The background fluorescence is excessive, making it difficult to discern the colocalization between endogenous RacM and macropinosomes. As this figure supports a key claim, enhancing image clarity is essential.

Response:

We thank the reviewer for this comment. Fig. S5F has been replaced with improved images of endogenous EhRacM in the parental strain (HM1:IMSS cl6). Alexa Fluor 488-conjugated secondary antibody was used to reduce background and improve signal detection. Arrowheads indicate EhRacM localization at putative nascent macropinosomes, and an arrow marks the pseudopod.

6. Motility assay analysis (CellPathfinder)
The explanation of the motility parameters requires clarification. Specifically, the rationale for including "total distance" as a relevant metric is unclear, given that it is similar between strains, whereas "confinement" and "net distance" differ. The authors should clarify how each parameter contributes to understanding motility behaviour.

Response:

© 2026. Published by The Company of Biologists under the terms of the Creative Commons Attribution License (https://creativecommons.org/licenses/by/4.0/).

We thank the reviewer for this comment. Total distance was originally included to describe overall movement, whereas net distance reflects displacement and the confinement ratio (net distance/total distance) indicates directional persistence. To improve clarity, we now focus on average speed and confinement ratio as the primary motility parameters, as these parameters more directly capture differences in migration behavior between strains. Definitions and analysis procedures are referenced in our previous study (Shimoyama et al., 2024).

References

Shimoyama, M., Nakada-Tsukui, K., Nozaki, T., 2024. EhRacM differentially regulates macropinocytosis and motility in the enteric protozoan parasite *Entamoeba histolytica*. PLoS Pathog. 20, e1012364.

Welter, B.H., Powell, R.R., Laughlin, R.C., McGugan, G.C., Bonner, M., King, A., Temesvari, L.A., 2006. *Entamoeba histolytica*: comparison of the role of receptors and filamentous actin among various endocytic processes. Exp. Parasitol. 113, 91–99.

Second decision letter

MS ID#: jcs.264490R1

MS Title: A RhoGEF activates EhRacM to selectively promote motility without affecting macropinocytosis in Entamoeba histolytica

Authors: Misato Shimoyama; Kumiko Nakada-Tsukui; Tomoyoshi Nozaki
Article Type: Research Article

Dear Dr Nozaki,

I am happy to tell you that your manuscript has been accepted for publication in Journal of Cell Science, pending standard publication integrity checks.

© 2026. Published by The Company of Biologists under the terms of the Creative Commons Attribution License (https://creativecommons.org/licenses/by/4.0/).
